# Engineering a Functional Histidine Brace Copper-Binding Site into a *De Novo*-Designed Protein Scaffold

**DOI:** 10.1021/jacsau.5c00754

**Published:** 2025-09-29

**Authors:** Salvatore La Gatta, Linda Leone, Gianmattia Sgueglia, Lorena Šimunić, Yu-Kai Liao, Ondřej Vaněk, Marco Chino, Flavia Nastri, Mario Chiesa, Angela Lombardi

**Affiliations:** † Department of Chemical Sciences, University of Napoli Federico II, Via Cintia 26, 80126 Napoli, Italy; ‡ Department of Chemistry, University of Torino, Via Giuria 9, 10125-Torino, Italy; § Department of Biochemistry, Faculty of Science, 112302 Charles University, Hlavova 2030/8, 12800 Prague, Czech Republic

**Keywords:** *de novo* protein design, histidine brace copper-binding motif, lytic polysaccharide monooxygenase model, spectroscopic characterization, catalysis

## Abstract

*De novo* metalloprotein design has contributed to tremendous advances in bioinorganic chemistry by allowing the manufacturing of proteins with unique structures and functionalities that go beyond evolutionary constraints. Among the array of metal sites that can be engineered within *de novo* scaffolds, the design of catalytic copper centers is particularly challenging but still harder to achieve due to the versatile coordination environment and redox properties of the copper ion. Here, we present miniLPMO, a fully *de novo* protein, incorporating a functional histidine brace copper-binding site. Starting from a four-helix-bundle scaffold based on the designed homodimeric α_2_D protein, our design has integrated rational and computational strategies to optimize coordination shell residues. Circular dichroism and analytical ultracentrifugation experiments indicate that the folding and dimerization state is driven by copper binding. A detailed characterization by UV–Vis and EPR revealed that miniLPMO replicates the spectroscopic features of natural histidine brace sites. Finally, the designed metalloprotein catalyzes the cleavage of glycosidic bonds upon hydrogen peroxide activation, mimicking the activity of natural lytic polysaccharide monooxygenases (LPMOs). This study establishes the feasibility of integrating peculiar catalytic metal-binding sites into scaffolds unrelated to the native protein and designed entirely from scratch.

## Graphical abstract

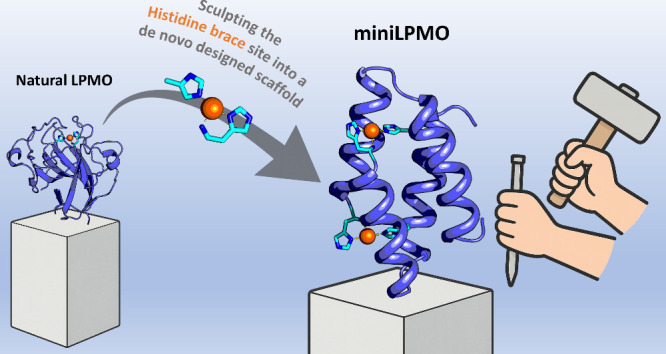

## Introduction

The variety of activities natural metalloproteins perform originates from the successful marriage between metal ions and protein scaffolds.^1^ However, they are limited by evolutionary constraints, which offer a finite array of protein folds and metal-binding sites tailored for specific biological roles. *De novo* metalloprotein design, involving the construction of metalloproteins “from scratch”,^2–5^ enables researchers to overcome the evolutionary boundaries by crafting novel metalloproteins with structures and functions still unexplored by nature. This approach can be regarded as a means to isolate and examine the active site of metalloproteins in a new, yet folded scaffold, allowing to dissect all interactions responsible for function. The power of this approach lies in the countless combinations of protein scaffold and metal-binding active sites that can be generated. In turn, this greatly expands the repertoire of functions beyond the capabilities of natural metalloproteins, delivering artificial biomolecules with potential applications in several fields, including catalysis,^6–15^ biosensing,^16,17^ and bioremediation.^18–20^

Numerous metal-binding sites have been engineered into *de novo* scaffolds, spanning different levels of designability.^21^ Heme centers are undoubtedly the most straightforward coordination site to reproduce within *de novo* systems because the porphyrin ligand provides most of the stabilization to the metal ion, while the peptide scaffold tunes its behavior by featuring the axial ligands and second shell residues.^1,22,23^ However, thanks to significant advances achieved in *de novo* metalloprotein design, it is now possible to precisely engineer sophisticated coordination environments, producing protein scaffolds able to host mononuclear,^14,15,24–28^ dinuclear,^29–32^ and multinuclear metal sites.^33–36^

Copper binding sites involved in C–H bond activation are fascinating targets for protein design due to their inherent ability to promote valuable transformations, such as the depolymerization of crystalline polysaccharides and methane oxidation.^37–39^ Among these, the histidine brace (HB), formally classified as a T2Cu center, is predominantly found in enzymes like lytic polysaccharide monooxygenases (LPMOs),^40–42^ where it plays a central catalytic role. Other HB motifs have also been found in copper-binding proteins such as CopC,^43,44^ which are involved in copper homeostasis and do not display oxidative activity. A similar HB motif has also been identified in particulate methane monooxygenases (pMMOs),^45,46^ although its catalytic relevance remains debated. Even though its function varies across proteins, the HB retains a characteristic structural identity within the broader family of copper binding sites in natural proteins.

All so far characterized LPMOs share a common structure formed by a core β-sandwich-like immunoglobulins, which accommodates the HB on the surface (Figure 1A). The copper coordination environment comprises an N-terminal His, which coordinates copper through both the amino terminus and the Nδ imidazole nitrogen. A second His binds copper through the Nε imidazole nitrogen, creating a tridentate T-shaped geometry (Figure 1B). Additionally, a Tyr residue and water molecules may, in some enzyme families, complete the first copper coordination shell, although the direct axial coordination of tyrosine is often ambiguous due to partial Cu^2+^ reduction during X-ray data collection or to substrate binding,^48^ which can shorten the Cu–O­(Tyr) bond.

**Figure 1:**
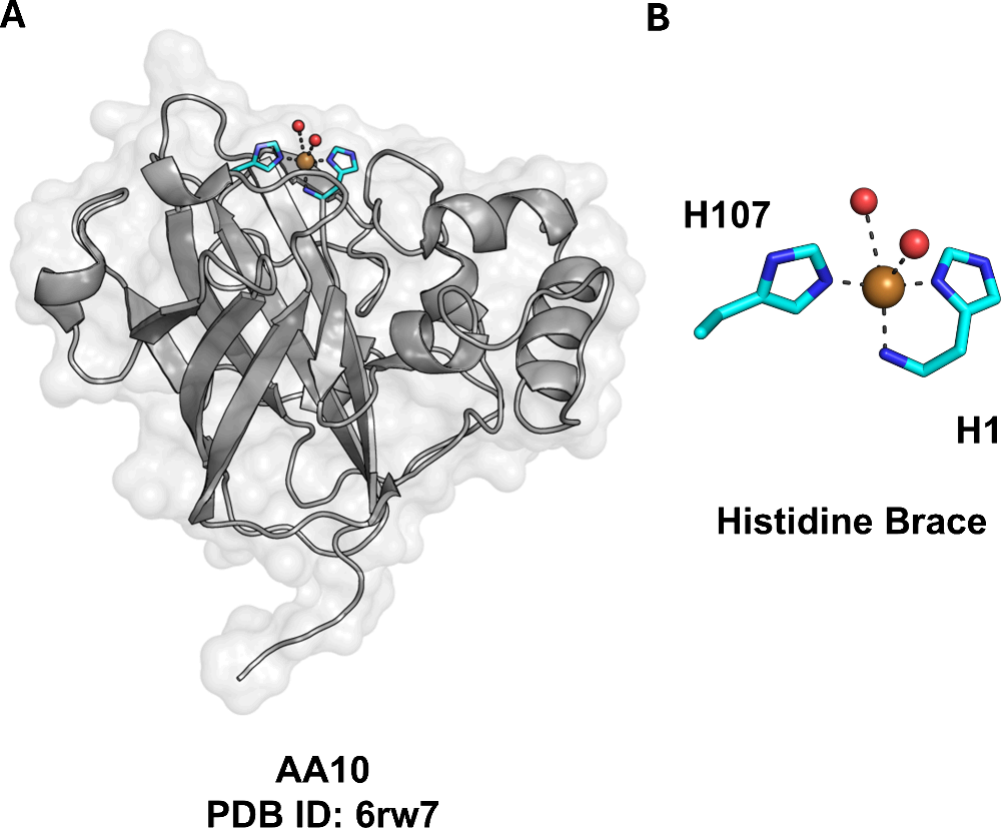
X-ray structure of a typical LPMO and its active site. (A) The crystal structure and (B) zoom of the active site of AA10 LPMO from *Teredinibacter turnerae* (PDB ID: 6rw7).^47^ The crystal structure is displayed in cartoon representation. The active site residues are shown as sticks with cyan-colored carbon atoms, the copper ion as a brown sphere and the water molecules as red spheres.

The most intriguing aspect of the HB site is the jarring contrast between the simplicity of this small structural unit and its incredible oxidizing power, which allows the conversion of substrates very resistant to oxidation.^49^ Indeed, starting from their discovery in 2011,^50,51^ we have seen an explosion of research dealing with LPMO structure–activity relationship studies.^52^ Nevertheless, several unresolved issues, such as the effect of second coordination shell interaction on the oxidative power of the Cu active site and the mechanism of substrate oxidation,^53^ deserve to be further addressed.

Reproducing the HB architecture in an artificial system capable of preserving its functionality represents a great challenge, in the context of LPMO application to biomass degradation. Several examples of LPMO mimics of different sizes and complexity have been recently reported. They span from small molecule ligands that recapitulate the LPMO coordination environment^54–58^ to short linear or cyclic peptide sequences emulating the natural amino-terminal copper and nickel binding (ATCUN) motif^59^ or minimalistic heterochiral tri- or penta-peptides resembling the HB active site.^60,61^

Although the HB mononuclear copper coordination site may appear simple to reproduce in model systems, there is considerable complexity outside the first coordination shell. Thus, artificial LMPOs have also been obtained by handling ligands with increasing levels of complexity around the metal site. Recent examples deal with the modification of the natural electron transfer protein azurin to house a functional HB site^62^ and the crafting of a small-molecule Cu biotinylated-complex into streptavidin, employing the biotin–streptavidin methodology.^63^ We approached this challenge by using *de novo* protein design. Our group has previously developed several peptide-based metalloenzymes, representing a middle ground between small molecule mimetics and large native proteins. By *de novo* design, we successfully reproduced the structural and functional features of metalloenzymes, housing heme,^8,12,19,64,65^ iron–sulfur centers,^66^ and dinuclear cofactors as diiron and dicopper centers.^30,32,67^

Recent advances in machine learning methodologies have proven their invaluable contribution to computational protein design.^68^ However, the *de novo* design of a predefined arrangement of primary and secondary coordination shells around a metal cofactor still represents a challenging endeavor. Especially in the case of HB, the peculiar N-terminal His ligand defies the current capabilities of state-of-the-art (SOTA) structure prediction models (e.g., Alphafold3 and Chai-1).^69,70^

Herein, we showcase the results on miniLPMO, a *de novo* metalloenzyme hosting a functional HB copper-binding site. Our design strategy combines rational design with an established pipeline^66,67^ involving computational tools that aim at stabilizing the desired cofactor into a predefined miniaturized scaffold. In particular, we devised to implant the HB site into a four-helix bundle scaffold, a protein fold entirely different from its natural counterparts. We selected such a scaffold for its exceptional stability, which offers the opportunity to screen multiple substitutions. Our model was based on α_2_D,^71,72^ a designed protein featuring a peculiar bisecting U topology. Such bisecting U motif, first identified in α_2_D by DeGrado and co-workers, was then recognized as a recurring folding motif in natural proteins.^72,73^ A detailed characterization revealed that miniLPMO hosts an HB copper-binding site at neutral pH, closely matching the spectroscopic features of the natural counterparts. Further, preliminary catalytic studies showed that the artificial enzyme promotes the cleavage of glycosidic bonds upon activation of hydrogen peroxide (H_2_O_2_), thus behaving as a functional mimic of LPMOs.

## Results and Discussion

### miniLPMO Design

In natural LPMOs, the HB site is found on the protein surface. Proximity analysis of its structural context in representative crystal structures of LPMOs (Table S1)^47^ points out that 13 to 15 residues fall within an 8 Å sphere around the copper ion (Figure 2A and Figure S1, Figure S2) as compared to the 18 residues neighboring the more buried type II copper site in *H. rosellus* galactose oxidase (PDB ID: 1gof). With this in mind, we aimed to select the best topology able to expose the metal site on the surface, still being capable of supporting its activity with a set of second shell interactions.

**Figure 2:**
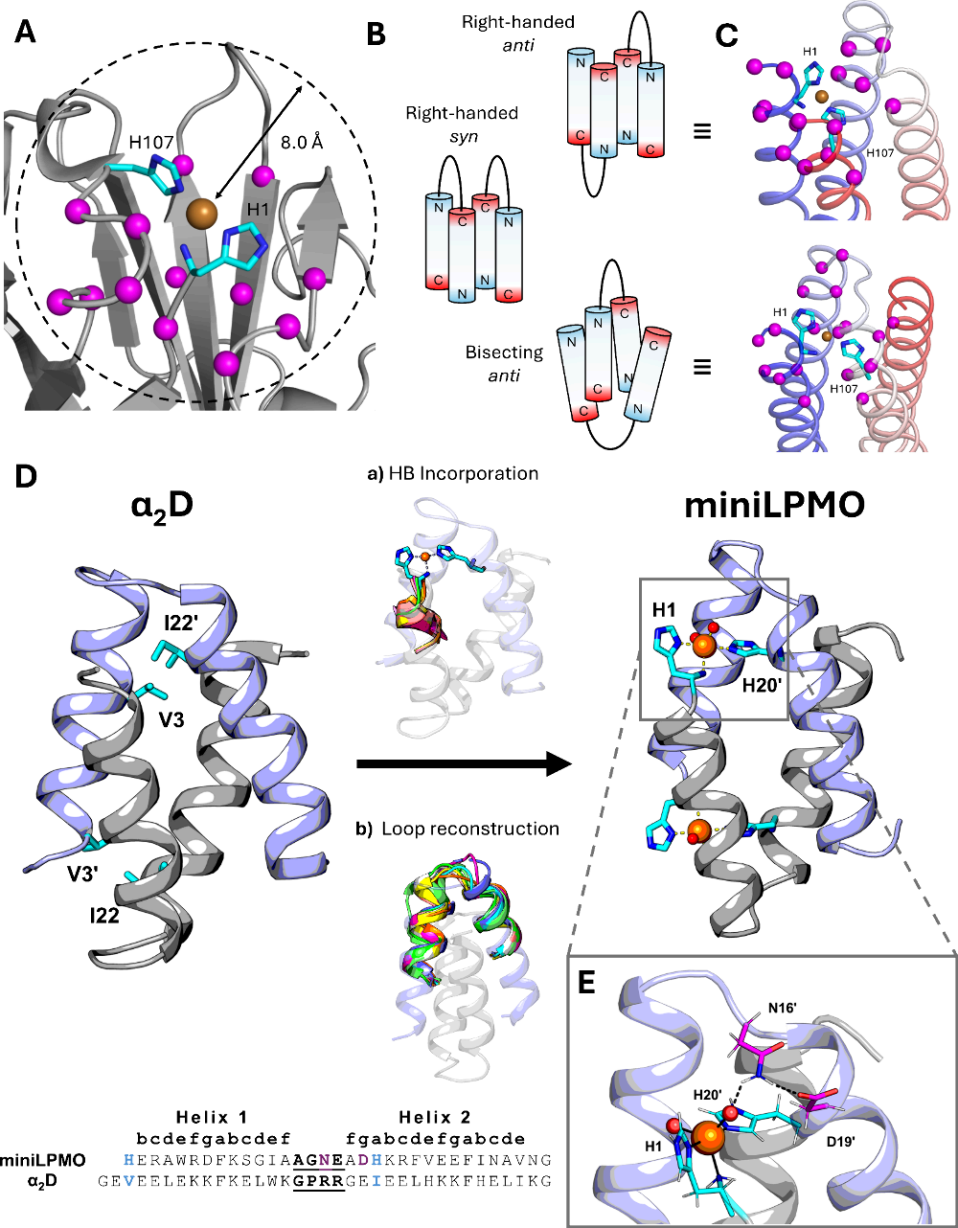
Design strategy of miniLPMO. (A) Target metal site for the design of miniLPMO located in AA10 from *Teredinibacter turnerae* (PDB ID: 6rw7) with C-α atoms of residues within 8.0 Å of the copper atom represented as magenta spheres. (B) Possible topologies of homodimeric 4-helix bundle proteins. Only the clockwise turning bundles are shown for simplicity. (C) Comparison of computationally generated homodimeric 4-helix bundle proteins with anti and bisecting U bundle topologies. The HB site of *T. turnerae* AA10 (cyan) is placed in the two bundles by superimposing the N-terminal His residue to the first N-terminal residue facing the interior of the bundle. (D) Design strategy of miniLPMO involving (a) HB incorporation and (b) loop reconstruction in the original α_2_D scaffold. The two symmetry-related α_2_ motifs are represented in different colors (gray and violet) for clarity of display. In the peptide sequence alignment of miniLPMO with α_2_D the coordinating residues are reported in cyan, the loop residues in underlined bold, and residues involved in second shell interactions in purple. (E) Close-up of the hydrogen-bond network surrounding the metal-bound water molecules in the final miniLPMO model.

We have previously designed homo- and heterodimeric antiparallel four-helix bundles, able to accommodate dinuclear, functional metal sites.^6,30,32,67^ The intrinsic advantage of this scaffold is its ability to achieve considerable topological complexity with a minimal sequence length.^73^ Compared to single-chain and four-chain architectures, the dimeric scaffold exhibits six possible topologies (Figure 2B):^73^ three loop arrangements (left-handed, right-handed, and bisecting U), each occurring in *syn* or *anti* orientations based on whether the connecting loops are on the same or opposite sides of the bundle. We parametrically built^74^ and evaluated the topologies based on their ability to accommodate the metal-binding site at the N-terminus while maintaining an optimal number of stabilizing interactions (see Table S1). The *anti*-bisecting topology, where the connecting loop traverses the bundle diagonally, emerged as the optimal configuration (Figure 2C). This arrangement allows favorable packing interactions between the N-terminal helix and the diagonal loop, while avoiding potential steric clashes or destabilizing interactions that could arise from a proximal flexible C-terminal helix. Only a few examples of bisecting four helical bundles of either designed^71,75^ or natural proteins^76–79^ were present in the PDB. Among them, the smaller and most compact scaffold is α_2_D, a *de novo*-designed 35-residue helix–loop–helix peptide.^71^ Therefore, we selected α_2_D as a blueprint for the design of miniLPMO.

Using the α_2_D NMR solution structure (PDB ID: 1qp6)^72^ (Figure 2D), the design started by selecting the best position in the template to allocate the internal His of the HB site. To this end, all possible backbone-dependent rotamers for this residue were superimposed on the α_2_D residues of one monomer located within 10 Å of the N-terminus of the other monomer. Ile to His substitution at position 22 (Figure 2D) appears suitable as it places His in helix 2 facing position 3′ (Figure 2D, numbers with prime refer to symmetry-related residues in the symmetric dimer) of the helix 1’ upon homodimer formation. Therefore, removing the first two residues from the N-termini and Val3His substitution would allow HB reconstruction in α_2_D with minimal backbone rearrangement (Figure 2D). Next, a backbone geometry optimization for the terminal His position was performed using the MASTER (*Method of Accelerated Search for Tertiary Ensemble Representatives*) software (see Figure S3A). The loop region was also reconstructed for the following reasons: (i) the removal of the two initial residues from α_2_D causes the loop to be more exposed to the solvent with respect to the starting structure; (ii) the loop region is sufficiently close to the HB metal-binding site, and thus it may influence the metal site structure, both in terms of steric hindrance and primary/secondary coordination shells. Loop reconstruction was performed using MASTER, which generated fragments to fill the gap in a query structure formed by two helix segments taken from the α_2_D monomer (see Figure S3B). The best matches (Figure 2D) were mostly similar in backbone geometry, and one match seemed particularly appropriate, thanks to the presence of a Gln residue in position 16. Such residue is close enough to the metal site, thus resembling the Gln166 or Glu193 homologous side chains, often found in the second coordination shell of natural LPMOs (PDB ID: 7t5d, 6rw7, Figure S2). Residues in these positions appear to be implicated in hydrogen bonding to either O_2_ or H_2_O_2_ molecules during the catalytic cycle and is probably also important for specific interactions with the substrate polysaccharide.^38,80^ Finally, the backbone coordinates so far obtained were employed as a starting point for computational design through the ROSETTA software suite,^81^ yielding a newly designed sequence unrelated to the parent α_2_D (see Supporting Information).

As a further validation method for the designed structure, MD simulations were performed on the lowest energy sequence, as quantified by the Ref2015 score function in ROSETTA,^82^ and on a subset of mutants in positions 16 and 19. Zn^2+^ was used as a proxy of Cu^2+^ for the MD simulation, as previously reported.^32^ In addition, zinc substitution in natural LPMOs does not significantly alter the orientation of the first coordination shell.^83^ Coordination shell analysis of homodimers during MD simulations suggested that long side chain coordinating residues (such as Glu and Gln) should be avoided in positions 16 and 19 (see Figure S4). Indeed, shorter side chain residues were preferred to avoid undesired coordination to the metal (Figure 2E), but still able to form a hydrogen bond with either water or reactive oxygen-bound intermediates in the first coordination shell, as occurring in natural LPMOs (see Figure S2, position 193). Asn in position 16 and Asp in position 19 were chosen, and the final model of miniLPMO was obtained. This sequence was subsequently synthesized and characterized.

### miniLPMO Folding and Oligomerization State

miniLPMO sequence was synthesized by solid-phase peptide synthesis, purified to homogeneity by RP-HPLC, and identified by ESI-MS (see Figure S5 and Supporting Information for experimental details).

The folding of apo-miniLPMO was first assayed by circular dichroism (CD) analysis at different concentrations and pHs (Figure S6). At pH 7.5 and at low peptide concentration (25 μM), apo-miniLPMO shows low helicity, as the CD spectrum displays a strong negative band at 201 nm and a shoulder at 225 nm (Figure S6A). Upon increasing peptide concentration up to 100 μM, conformational changes were reflected in the CD spectrum, as the low-wavelength minimum shifted toward 207 nm, and the mean residue ellipticity (MRE) at 222 nm increased (Figure S6A). Increasing of helical content was also observed raising the pH from 4.5 to 11 at 100 μM peptide concentration (Figure S6B). Addition of stoichiometric amount of Cu^2+^ to apo-miniLPMO led to a small increase in the helical content, as highlighted by the comparison of CD spectra of the apo- and the holo- forms at 100 μM, at neutral pH (Figure 3A). Indeed, a small increase in the θ_ratio_ (θ_222 nm_/θ_205 nm_) from 0.54 in the apo to 0.61 in the holo form was observed, together with an increased ellipticity at 190 nm. Inspection of Figure S7 shows that this behavior occurs at both acidic and neutral pH conditions, suggesting that peptide folding and assembly could be assisted by copper binding. At pH 11, where a more regular helical folding is observed in the apo form, no significant changes where observed upon copper addition.

**Figure 3:**
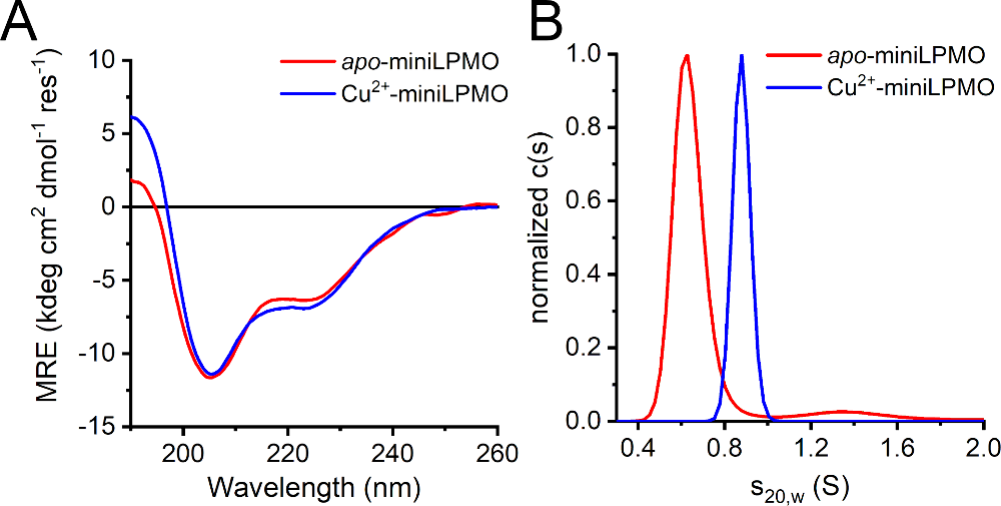
Folding and oligomerization state of miniLPMO. (A) Far-UV CD spectra of miniLPMO (100 μM in 5 mM MES, 5 mM HEPES, 5 mM CHES pH 7.4) in the absence (red line) or presence (blue line) of 100 μM CuSO_4_. (B) Sedimentation analysis of 100 μM apo- (red line) and holo-miniLPMO (blue line) in 30 mM HEPES buffer pH 7.5.

Based on these findings, the role of copper in miniLPMO assembly and oligomerization was investigated by analytical ultracentrifugation (AUC). The apo- and holo- forms were initially analyzed in three different pH conditions (pH 4.5, 6.5 and 9.5) at 80 μM concentration (Figure S8A,B). The apo-miniLPMO sedimented with weight-average sedimentation coefficient s_20,w_ value of 0.65 S across the whole pH range, corresponding to a species with a predicted molecular weight (MW) of 3.6–4.2 kDa, matching well the theoretical MW of monomeric miniLPMO (3.77 kDa). The fitted frictional coefficients’ ratio f/f_0_ of 1.45–1.55 points to an elongated molecule shape, possibly an extended conformation of the helical peptide. The same behavior was observed for holo-miniLPMO at pH 4.5 (at both 80 mM and 300 mM, Figure S8B). Interestingly, at pH 6.5 and 9.5, it sedimented with an average s_20,w_ of 0.95 S and f/f_0_ of 1.4–1.7, corresponding to a predicted MW of 6.5–8.3 kDa, suggesting the presence of the expected dimeric species (MW 7.67 kDa). Next, focusing on the neutral pH range, the apo- and holo-miniLPMO were analyzed at various concentrations at pH 7.5 (Figure S8C–E). The apo-miniLPMO sedimented primarily as a monomer with s_20,w_ of 0.65–0.7 S (Figure S8C,D) at low peptide concentration. However, upon increasing its concentration from 25 to 1000 μM, a second species with s_20,w_ of 1.4–1.5 S was observed, which, taken together with the overall f/f_0_ values of 1.4–1.7, corresponds to MW of 13.5–16.5 kDa, best explained by a tetramer. On the other hand, using peptide concentrations ranging from 25 to 100 μM, the holo-miniLPMO sedimented as a dimer with s_20,w_ of 0.94 S (Figure S8E), supported by the f/f_0_ values of 1.4–1.5 leading to predicted MW of 6.5–7.0 kDa. Unfortunately, analysis of the holo form at higher concentrations was hampered by precipitation. The comparison of sedimentation profiles at 100 μM and pH 7.5 of apo- and holo-miniLPMO (Figure 3B) clearly indicates that copper binding favors the dimerization in these experimental conditions. Compared with the s_20,w_ and f/f_0_ values of 1.23 S and 1.12, respectively, calculated for the α_2_D globular dimer (PDB ID: 1qp6) using the program HullRad,^84^ sedimentation analysis suggests that miniLPMO adopts less globular, more extended conformations in solution, both as a monomer and as a dimer.

### Cu^2+^ and Cu^+^ Binding Affinities

Having ascertained the presence of the expected holo-dimeric species at neutral pH and peptide concentrations ranging from 25 to 100 μM, we investigated whether miniLPMO could form stable complexes with copper in both Cu^2+^ and Cu^+^ oxidation states. The affinity of miniLPMO toward Cu^2+^ was studied by spectrofluorimetric titrations of the apoprotein with CuSO_4_, following quenching of Trp fluorescence upon Cu^2+^ binding (see Supporting Information for experimental details). Experimental data were fitted to a 1:1 binding isotherm, assuming the intended Cu^2+^:monomer ratio of 1:1 and giving an apparent K_d_ of (4 ± 1) × 10^–10^ M at pH 7 (Figure S9). The dissociation constant value found for the Cu^2+^-miniLPMO complex is in the nanomolar range, in line with those reported for other *de novo* metalloproteins hosting type 2 copper centers.^85,86^ It is worth noting that the presence of a monomer–dimer equilibrium for miniLPMO may affect the copper binding affinity especially at the low concentrations used in fluorescence experiments.

As a functional copper site requires the ability to undergo redox cycling, the affinity of miniLPMO toward Cu^+^ was also analyzed. The dissociation constant of the Cu^+^-miniLPMO complex was determined by spectrophotometric titrations using bicinchoninic acid (BCA) as a competitive ligand for Cu^+^ ions, following the formation of the [Cu^+^(BCA)_2_]^3–^ complex^87^ (see Supporting Information for experimental details). Fitting of experimental data to a ligand competition equilibrium (see eq 2 of Supporting Information and Figure S10) gave a K_d_ value of (2.1 ± 0.2) × 10^–12^ M at pH 7, in line with those reported in the literature for similar systems.^86^ The reduction potential was estimated based on the Nernst equation (see Supporting Information eq 3) using the values of K_d_ found for Cu^2+^-miniLPMO and Cu^+^-miniLPMO complexes. The calculated reduction potential for the Cu^2+^/Cu^+^-miniLPMO couple was 291 ± 11 mV (vs SHE), which is close to those of natural LPMOs,^88,89^ and in the range of other *de novo*-designed type 2 copper proteins.^85,90^

### Spectroscopic Characterization of the Copper Center

The first coordination shell of the HB site in the miniLPMO model incorporates ligands with ionizable protons, such as histidine residues, terminal amine, and water molecules. Thus, changes in the copper coordination state occur as a function of pH, as observed for natural LPMOs.^91–93^ To gain a complete spectroscopic characterization of the copper site, UV–Vis and X-band Continuous Wave Electron Paramagnetic Resonance (CW-EPR) spectra were recorded in the 4.5–11 pH range (Figure S11).

A progressive blue-shift of the *d-d* bands and changes in the EPR spectra (Figure 4 and Figure S11) are observed upon pH raising. In particular, a pronounced change is observed at pH 8.5, indicating that a change in the copper coordination environment occurs at this pH.

**Figure 4:**
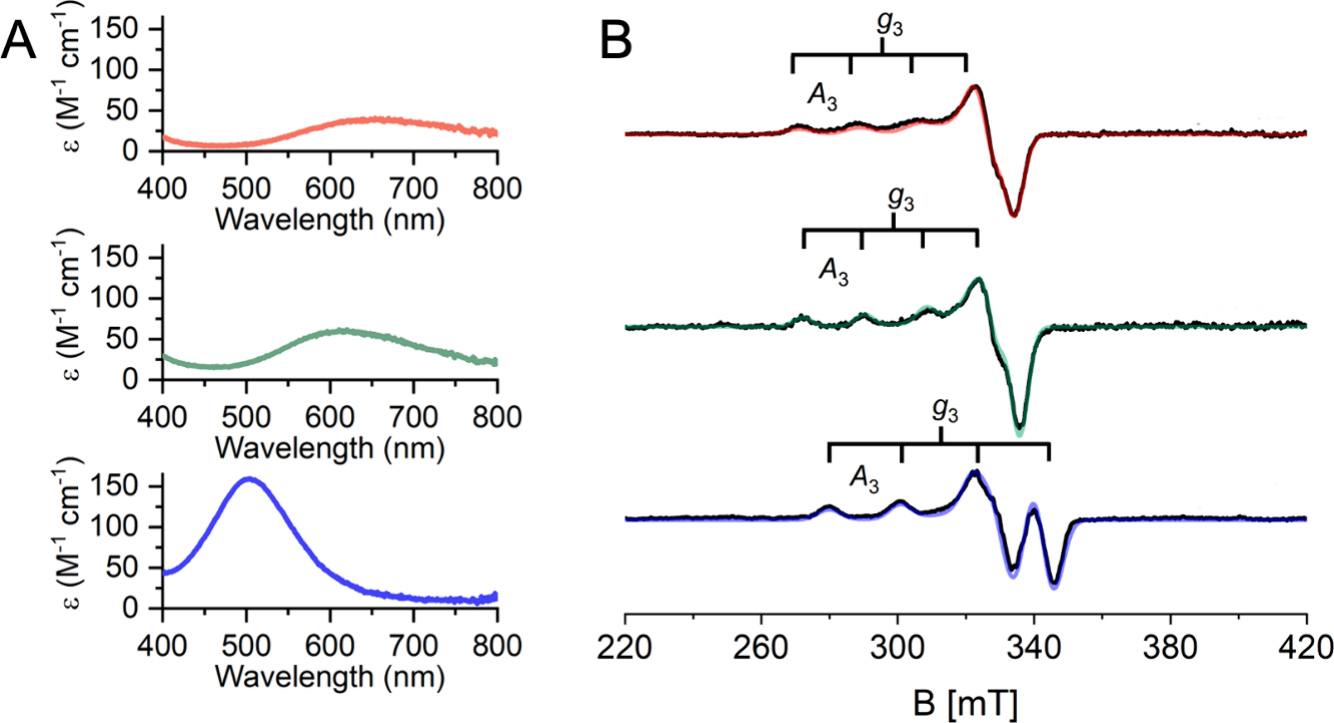
Spectroscopic studies of Cu^2+^-miniLPMO. (A) Visible absorption spectra of Cu^2+^-miniLPMO (200 μM) acquired at room temperature and (B) X-band CW-EPR spectra of Cu^2+^-miniLPMO (300 μM) recorded at 77 K at pH 4.5 (red traces), pH 7.0 (green traces), and pH 11 (blue traces).

At low to neutral pH, the EPR spectra (Figure 4B and Figure S11) show only minor changes and can be simulated assuming a single Cu^2+^ species with a nearly axial *g*-tensor (Table 1). In contrast, natural LPMOs typically exhibit more rhombic *g*- and ^Cu^ *A*-tensors, consistent with lower symmetry coordination sites in this pH range.^91,94^ At both pH 4.5 and 7, the *g*- and ^Cu^ *A*-parameters align with a singly occupied molecular orbital (SOMO) of dominant *d*(x^2^ – y^2^) character and a 2N2O ligand coordination environment, as classified in the canonical Peisach–Blumberg plot.^95,96^ A slight decrease in *g*_3_ and a corresponding increase in *A*_3_ are observed when moving from pH 4.5 to pH 7 (Table 1), suggesting minor changes in the local ligand field, possibly due to geometric distortions. Even subtle alterations in imidazole coordination can modulate electron density delocalization between copper and the imidazole, thereby affecting the spin density on copper and resulting in shifts in the sensitive *g*_3_ and *A*_3_ parameters.^97^ These EPR observations are corroborated by visible absorption spectra in the same pH range, which show a modest blue shift in the *d-d* transition band and an increase in molar extinction coefficient (λ = 650 nm vs 620 nm; ε = 50 M^–1^ cm^–1^ vs 60 M^–1^ cm^–1^). Overall, both EPR and visible absorption data suggest a Cu^2+^ coordination environment featuring two directly ligated nitrogen atoms, characteristic of a Cu­(His)_2_ center with bound water molecules, at both pH values.^98^

**Table 1:** Spin Hamiltonian Parameters and *d-d* Electronic Absorption Transition of the pH Dependent Species^a^

Species (pH)	*g*_1_	*g*_2_	*g*_3_	|*A*_1_|	|*A*_2_|	|*A*_3_|	λ_max_ (ε)
1 (4.5)	2.060	2.067	2.268	40	40	535	650 (50)
3 (7)	2.052	2.062	2.251	60	35	560	620 (60)
2 (11.0)	2.038	2.042	2.167	75	55	622	505 (150)

a Hyperfine tensor principal values are given in units of MHz. The estimated error in the determination of *A*_1_, *A*_2_ is ∼20 MHz, while for *A*_3_ is ∼15 MHz. The uncertainty in the *g* values is ± 0.003. λ_max_ of the *d-d* electronic transition is given in nm, while ε in M^–1^ cm^–1^.

At alkaline conditions (pH ≥ 8.5), a significant spectral change is observed in the EPR spectrum with decreased *g*_3_ and increased ^Cu^ *A*_3_, accompanied by a blue-shift of the *d-d* transition in the visible absorption spectrum of ≈ 120 nm as well as a 3-fold increase of the molar extinction coefficient (Figure 4A, Figure S11, and Table 1). This finding is indicative of a more asymmetric coordination, with a stronger ligand field. The spectrum is satisfactorily reproduced by simulation of a single copper species, in agreement with natural LPMOs’ values at alkaline pH and a richer nitrogen coordinating environment (3N1O).^91–93^

Notably, the formation of the pH-dependent species is reversible, considering that the visible spectral properties of the species at neutral pH were fully restored upon raising the pH to 11 and then back to 7 (Figure S12).

To obtain a more detailed description of the local coordination environment of the Cu^2+^ Q-band Electron Nuclear Double Resonance (ENDOR) and X-band HYperfine Sublevel CORrElation (HYSCORE) spectroscopies were employed. Spectra were recorded at pH 4.5 and pH 11 (Figures S13–S15). Unfortunately, the low solubility of Cu^2+^-miniLPMO at pH 7 prevented the pulse EPR investigation at this pH and a more detailed description of the small structural changes reported by CW-EPR and visible absorption spectra. The Q-band ENDOR spectrum of strongly coupled ^14^N nuclei is characterized by pairs of lines centered at half the hyperfine coupling (*A*) and split by twice the ^14^N nuclear Larmor frequency (ν_N_ ≈ 3.6 MHz). At low magnetic field the ENDOR spectrum probes a single crystal-like position out of all possible orientations^99–101^ and shows one single ENDOR doublet (N1 in Figure 5) with coupling of *A* ≈ 36 MHz (Figure 5). Simulation of the spectrum recorded at two magnetic field settings (Figure S13) indicates an axial ^14N^ *A* tensor (Table S2) with the largest coupling (*A* = 45 MHz) aligned along *g*_1_/*g*_2_ and pointing toward the Cu–N bond and a nuclear quadrupole coupling *e* ^2^ *qQ*/*h* ≈ 2.5 MHz consistent with values reported for proteins with histidine-coordinated copper centers^100–103^ and a recent ENDOR study on the AA10 LPMO *Sm*AA10A (CBP21).^93^ At high pH (pH 11), the single crystal-like ENDOR spectrum features an extra line at a lower frequency (10 MHz) and extends up to 26 MHz. The spectrum can be simulated assuming the presence of two sets of coordinated nitrogen ligands (N2 and N3 in Figure 5), with different couplings (Figure 5 and Figure S15). The full hyperfine and nuclear quadrupole tensors are estimated by simulating spectra recorded at different magnetic field settings (Supporting Information) and are listed in Table S2. The presence of a new ^14^N coupling with large *a*_iso_ (51 MHz) is tentatively assigned to the binding of a deprotonated amine function of the HB, as already observed in AA10 LPMOs.^91,93^ The coordination of the imidazole moieties at both pH values is confirmed by X-band ^14^N HYSCORE spectra (Figure 5, Figure S16), which show the presence of weakly coupled nitrogen nuclei with maximum coupling of the order of 2.4 MHz, characteristic of remote (“backside”) nitrogens of imidazole groups.^92^

**Figure 5:**
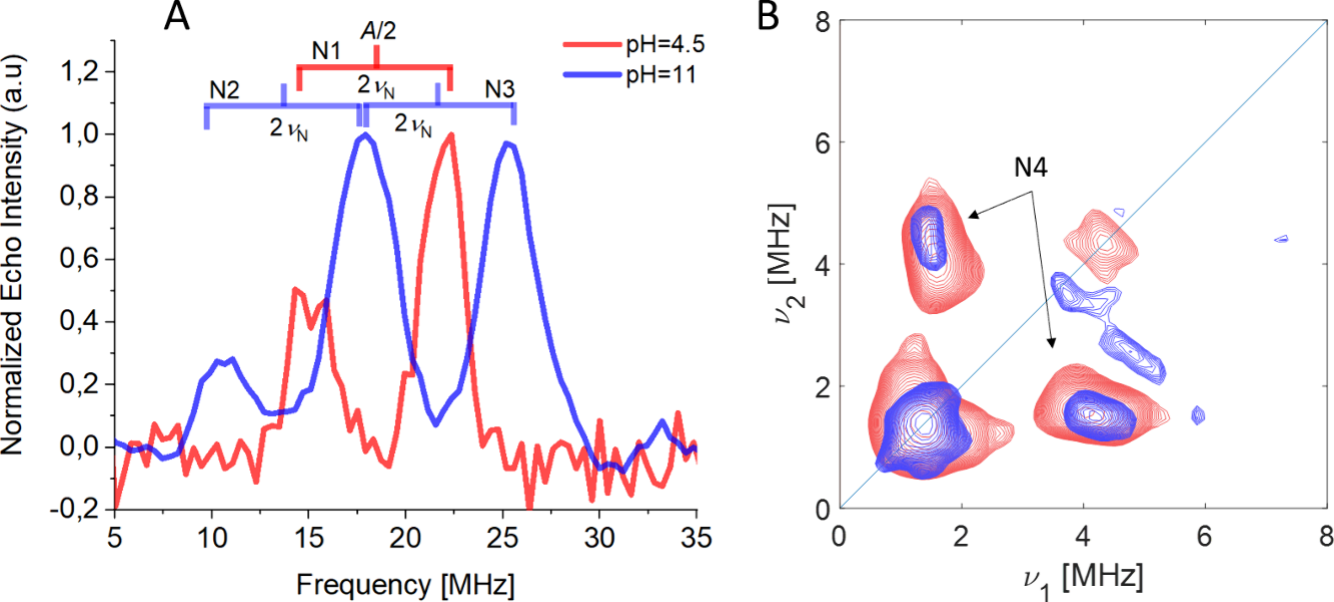
Pulsed EPR studies of Cu^2+^-LPMO. (A) Q-band Davies ^14^N ENDOR spectra (20 K) recorded at 1167.9 mT of species 1 (pH 4.5, red) and species 2 (pH 11 blue). (B) X-band 6p-HYSCORE spectra (30 K) of species 1 (red) and species 2 (blue), recorded at 336.8 mT.

Overall, the axial *g* and *A* tensors observed for the species at pH = 4.5 are in agreement with a tetragonally distorted octahedral environment with 2N2O axially coordinated ligands and a metal *d*(x^2^-y^2^) based SOMO. Based on the ENDOR and HYSCORE experiments, the coordinating nitrogen atoms are associated to two equivalent imidazole ligands as illustrated in Scheme 1. At alkaline pH, reduction of *g*_3_ and the increase in the *A*_3_^63^Cu hyperfine component and a blue shift of the absorption spectrum and appearance of a new large ^14^N coupling in the ENDOR spectrum indicate a change in the ligand field, consistent with the binding of a (deprotonated) amine of the HB (Scheme 1).

**Scheme 1:**
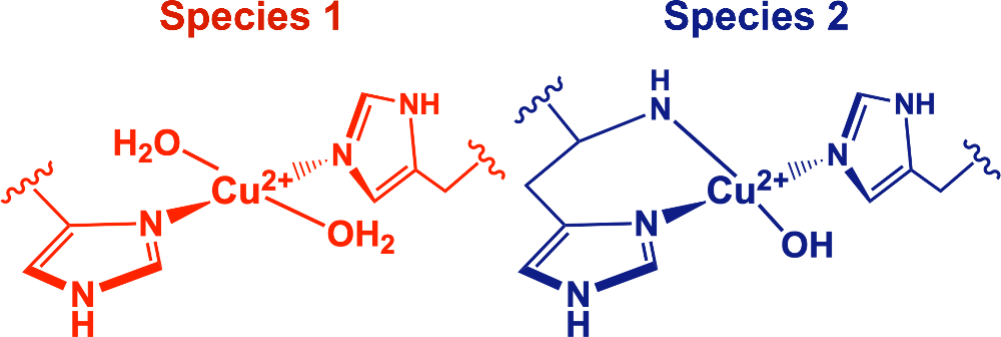
Schematic Equatorial Coordination Structure of the Copper Center in Cu^2+^-miniLPMO at Low and High pH

In conclusion, all the spectroscopic data obtained on the *de novo* miniLPMO protein show that the Cu^2+^ metal site coordinating environment is consistent with natural LPMO enzymes,^93^ and its response to pH changes allowed to identify two main species at pH 4.5 and pH 11 (Scheme 1), which both foresee a Cu­(His)_2_ center. Due to the strong interplay between the protein assembly and miniLPMO copper-binding site reconstruction, the EPR data can be reliably interpreted in view of the sedimentation analysis, which showed a pH-dependent oligomeric state of the holo-form, with a monomer prevalent at pH 4.5 and a dimer being formed from pH 6.5 (Figure S8B). Thus, the Cu­(His)_2_ center is consistent with a dimeric assembly at neutral to alkaline pHs, with one His derived from each monomer, as in the design. At pH 4.5, both histidines may derive from the same monomer, being the monomeric species prevalent at this pH up to 300 μM (Figure S8B). This finding is further supported by CD analysis at pH 4.5, where a lower helical folding was detected respect to neutral and alkaline pHs (Figures S6 and S7).

### Hydrogen Peroxide Activation by Cu^2+^-miniLPMO

To test the ability of the *de novo*-designed protein to act as a functional mimic of the HB motif in natural LPMOs, we performed preliminary activity assays. The oxidative activity of natural LPMOs depends on the activation of O_2_ and H_2_O_2_.^104,105^ The HB copper-binding site plays a pivotal role, interacting with these small molecules to form several copper–oxygen species, which, in turn, oxidize polysaccharide substrates. Even though the reaction with H_2_O_2_ can be deleterious to the enzyme,^106,107^ it provides a shunt that allows a faster formation of the reactive copper–oxygen species. Accordingly, we first investigated the ability of Cu^2+^-miniLPMO to bind and activate H_2_O_2_ at pH 7.8. The reaction of H_2_O_2_ with Cu^2+^-miniLPMO (100 μM) was monitored by following changes in the UV–Vis spectra, showing the appearance of an intense absorption at 305 nm. Interestingly, an absorption band at about 390 nm was also formed (Figure S17). This feature is typical of [Cu-OOH]^+^ species, assignable to the ligand to metal charge transfer transition from the OOH^–^ group to Cu^2+^, as already observed in a natural LPMO^108^ and small molecule mimics.^54,61,63^

Next, the oxidative reactivity of Cu^2+^-miniLPMO was evaluated in the C–H bond activation, using *p*-nitrophenyl-β-d-glucopyranoside (PNPG) as model substrate and H_2_O_2_ as cosubstrate (Figure 6A). The reaction was started by adding H_2_O_2_ to a solution containing Cu^2+^-miniLPMO and PNPG, and UV–Vis spectra were recorded during the reaction. The appearance and increase over time of a band at 400 nm was observed and ascribed to *p*-nitrophenolate formation (Figure 6B). Conversion was measured by taking aliquots at different times during the reaction and diluting them in carbonate buffer (pH 10.5), which ensures that all produced *p*-nitrophenol (PNP) is in the *p*-nitrophenolate form, enabling quantification using its molar extinction coefficient at 405 nm. No substrate conversion was observed in the absence of H_2_O_2_, thus suggesting that Cu^2+^-miniLPMO promotes PNPG cleavage through an oxidative pathway. The initial rates and substrate conversion were dependent on pH, with activity increasing from pH 4.5 to 8.5. A substantial decrease in activity was instead observed at strongly alkaline conditions (pH 11) (Figure 6C). When compared to free Cu^2+^ at neutral pH (7), Cu^2+^-miniLPMO promotes PNPG oxidation with an approximately 3-fold higher initial rate (1.85 μM min^–1^ vs 0.65 μM min^–1^) and a 2.5-fold higher substrate conversion (13 μM vs 5 μM) after 40 min (Figure S18).

**Figure 6:**
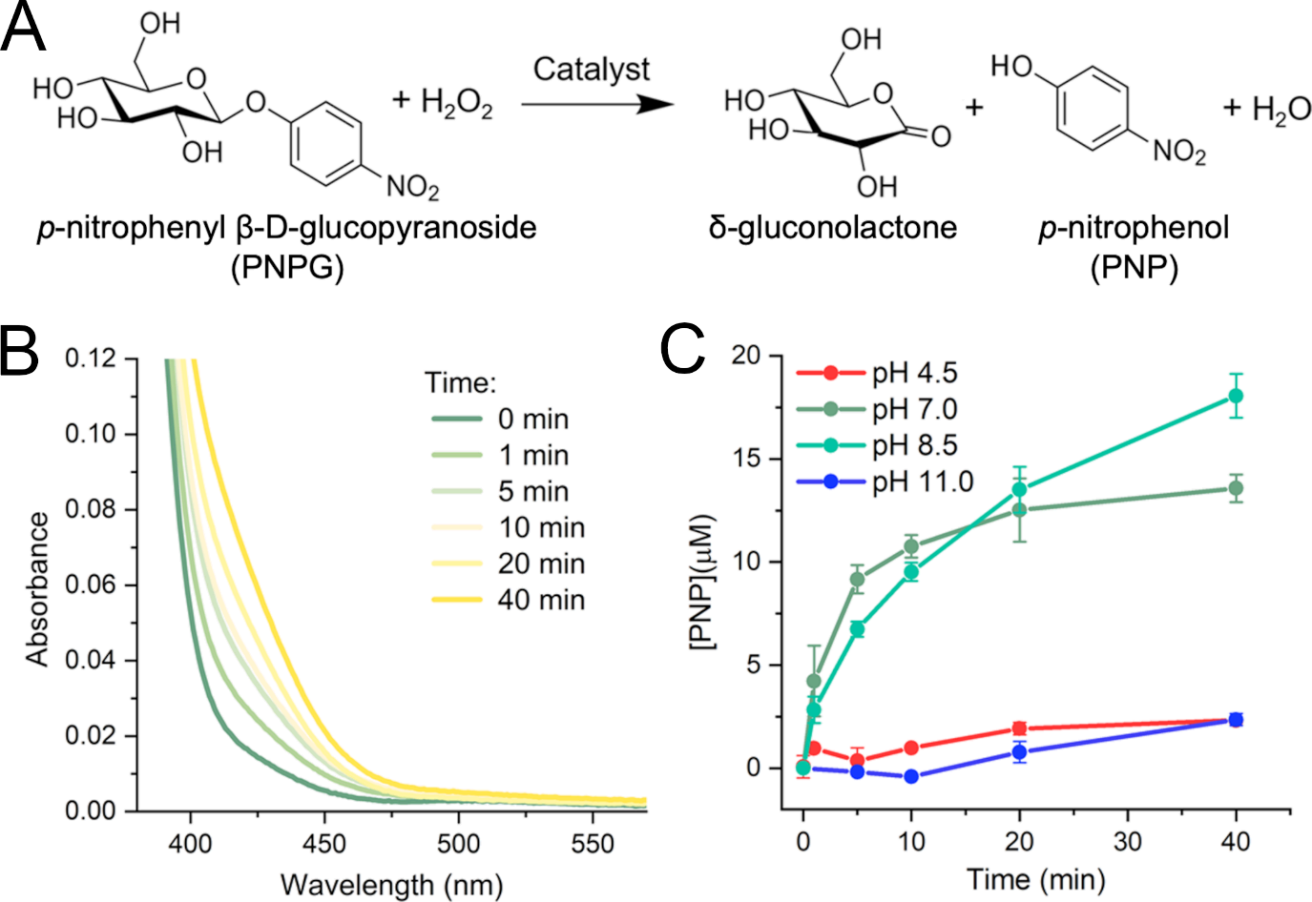
Catalytic studies of Cu^2+^-miniLPMO. (A) Reaction scheme of the oxidative cleavage of PNPG forming δ-gluconolactone and *p*-nitrophenol. (B) Visible spectra of the oxidation of PNPG catalyzed by Cu^2+^-miniLPMO at pH 7. (C) PNP formation kinetics using the different species of Cu^2+^-miniLPMO at different pHs: pH 4.5, pH 7, pH 8.5 and pH 11. The Cu^2+^-miniLPMO concentration was fixed at 80 μM, corresponding to the concentration of copper sites. PNPG concentration was fixed at 8 mM, in 15 mM MES, 15 mM HEPES, and 15 mM CHES as a buffer. The reaction was initiated by adding H_2_O_2_ to a final concentration of 80 mM. At fixed time intervals (circles), an aliquot of the solution was taken, 5-fold diluted in 100 mM carbonate buffer pH 10.5, and PNP concentration was monitored at 400 nm.

The observed increase in activity within the 4.5–8.5 pH range correlates well with the changes in the copper coordination environment. Indeed, the formation of the HB site begins at neutral pH, with a notable spectral shift occurring at pH 8.5. Although spectroscopic evidence indicates the presence of an HB site also at pH 11, the observed decline in reactivity is consistent with deprotonation of Cu^2+^ ligands (H_2_O to OH^–^ and N-terminal NH_2_ to NH^–^) at this pH. Indeed, the increase in the electron density at the copper center would stabilize the Cu^2+^ oxidation state and hinder the reduction to Cu^+^. This would hamper the copper ability to cycle between Cu^2+^ and Cu^+^, resulting in reduced catalytic activity at pH 11. Structural and kinetic constraints are probably the cause of the activity loss. Structural data by EPR spectroscopy are consistent with a square planar geometry with strong sigma-donor ligands, which saturates the equatorial coordination positions and may impose rigidity. This less flexible, highly coordinated environment may (i) limit H_2_O_2_ binding and (ii) hamper the coordination geometry changes necessary for Cu^2+^/Cu^+^ conversion, slowing electron-transfer kinetics.

## Conclusion

Inspired by the intriguing features of the HB copper-binding site of LPMOs, we have successfully engineered such motif into a *de novo*-designed peptide scaffold. As a starting point for the design of miniLPMO, we selected a *de novo* homodimeric four-helix bundle scaffold (α_2_D), which adopts the antibisecting U topology. The latter was preferred for optimizing the interactions between the N-terminal helix of one monomer with the loop of the other. Employing a combination of structure-based and computational design strategies, we identified the best positions for implanting the HB first and second coordination shell residues.

Our studies have shown that this miniprotein assumes the desired homodimeric assembly upon copper binding, forming stable complexes with copper in both Cu^2+^ and Cu^+^ oxidation states.

A detailed spectroscopic characterization through CW/pulsed-EPR and UV–Vis absorption spectroscopy demonstrated that the coordination environment of the copper-binding site in miniLPMO and its pH-dependent behavior are fully consistent with those of natural LPMO enzymes. To the best of our knowledge, *de novo*-designed metalloproteins featuring a HB copper binding-site have not been previously reported. Indeed, the few successful examples of HB design strictly rely either on the rigidity of the ligand moiety^60,63^ or on the preorganization of a natural protein scaffold.^62^ Remarkably, our model not only recapitulates the spectroscopic signature of the natural counterparts but was also found to activate H_2_O_2_, promoting the oxidative cleavage of a glycosidic bond in a model substrate at neutral pH. All herein reported results highlight the power of *de novo* design in developing artificial copper metalloenzymes, with potential implications in different fields. For instance, they may represent valuable tools for synthesizing complex molecules and producing high-value chemicals, in a sustainable and environmentally friendly manner. Overall, this study underscores the potential of this approach in developing functional metalloproteins that incorporate new and less explored metal sites.

## Experimental Section

The miniLPMO peptide was synthesized by automatic solid-phase synthesis using standard Fmoc protocols. To preserve the N-terminus free, it was not acetylated, whereas the C-terminus was amidated instead. It was purified to homogeneity via RP-HPLC, and its identity was ascertained by high-resolution ESI-MS (Figure S5). The concentration of miniLPMO is referred to the monomer throughout the text unless otherwise stated. CD measurements were performed using a J-1500 spectropolarimeter equipped with a thermostatic cell holder (JASCO, Easton, MD, USA). CD spectra were collected at 25 °C, from 260 to 190 at 0.5 nm intervals. UV–Vis absorption spectra were recorded with a Cary Varian 60 spectrophotometer equipped with a thermostatic cell compartment (Varian, Palo Alto, CA, USA) using a quartz cuvette with a 1 cm path length. Fluorescence experiments were performed on Fluoromax4 (Horiba Scientific). X-band (∼9.44 GHz) CW-EPR experiments were performed on a Bruker EMX spectrometer equipped with a cylindrical cavity. All spectra were recorded at 77 K and a microwave power of 0.68 mW, a modulation amplitude of 0.5 mT, and a modulation frequency of 100 kHz. Q-band Davies ENDOR measurements and six-pulse X-band HYSCORE experiments were performed on a Bruker Elexsys E580 spectrometer at 30 K. All of the EPR, ENDOR, and HYSCORE simulations were performed using the Easyspin software package.^109^

Sedimentation analysis, used to monitor the oligomeric state and associative behavior of biomacromolecules,^110,111^ was performed on the analytical ultracentrifuge ProteomeLab XL-I (Beckman Coulter, Brea, CA, USA) using an An50-Ti rotor and double-sector cells equipped with 1.5, 3, or 12 mm titanium centerpieces (Nanolytics, Potsdam, Germany), depending on the sample absorbance.^112,113^ Buffer density, viscosity, and miniLPMO partial specific volume were estimated in Sednterp.^114^ Data were analyzed with Sedfit^115^ using the c(s) continuous sedimentation coefficient distribution model. Figures were prepared in GUSSI.^116^ Detailed procedures of the experiments are available in the Supporting Information.

## Supplementary Material



## Notes

Additional experimental details, peptide design, synthetic procedures, peptide analysis, spectroscopic data, Figures S1–S18, and Tables S1–S2 (PDF)

## ∇

Department of Chemistry, University of Michigan, Ann Arbor, MI 48109, USA
