# Supplementary material for: Engineering a Functional Histidine Brace Copper-Binding Site into a De Novo-Designed Protein Scaffold
Source: JACS Au. 2025 Sep 29;5(10):4799–810. doi: 10.1021/jacsau.5c00754 (PMC12569686; doi:10.1021/jacsau.5c00754)
Supplement: Supplementary file 1 [file au5c00754_si_001.pdf]

Supporting Information for:

# Engineering a Functional Histidine Brace Copper-Binding Site into a *De Novo*-Designed Protein Scaffold

*Salvatore La Gatta,[a]† Linda Leone,[a] Gianmattia Sgueglia,[a] Lorena Šimunić,[a] Yu-Kai Liao,[b] Ondřej Vaněk,[c] Marco Chino,[a] Flavia Nastri,[a] Mario Chiesa,[b,*] Angela Lombardi[a,*]*

[a] Department of Chemical Sciences, University of Napoli Federico II, Via Cintia 26, 80126 Napoli, Italy

[b] Department of Chemistry, University of Torino, Via Giuria 9, 10125-Torino, Italy

[c] Department of Biochemistry, Faculty of Science, Charles University, Hlavova 2030/8, 12800 Prague, Czech Republic

**Corresponding Authors:**

Angela Lombardi, email: alombard@unina.it; Mario Chiesa, email: mario.chiesa@unito.it

**Present Address**

†Department of Chemistry, University of Michigan, Ann Arbor, MI 48109, USA.

**Supporting Information text**

Design of miniLPMO ........................................ 2
Peptide synthesis and purification ........................................ 4
UV-Vis spectroscopy ........................................ 5
CD spectroscopy ........................................ 5
Analytical Ultracentrifugation (AUC) ........................................ 6
$Cu^{2+}$ and $Cu^{+}$ binding affinities ........................................ 6
EPR spectroscopy ........................................ 8
Hydrogen peroxide activation ........................................ 9
*p*-nitrophenyl-β-D-glucopyranoside (PNPG) oxidation ........................................ 9

**Figures S1 to S14**

Fig. S1. Distribution of neighboring residue number among LPMOs ........................................ 11
Fig. S2. Logo representing amino acid conservation of the 15 neighboring residues .... 12
Fig. S3. Redesign of N-terminal segment and loop in $\alpha_2$D ........................................ 13
Fig. S4. MD simulations on miniLPMO variants generated by Rosetta ........................................ 14
Fig. S5. RP-HPLC chromatogram and ESI-MS spectrum of pure miniLPMO ........................................ 15
Fig. S6. CD spectra of apo-miniLPMO at different concentrations and different pHs .. 16
Fig. S7 CD spectra of apo- and holo-miniLPMO at different pH values ........................................ 17
Fig. S8. Sedimentation velocity analysis of miniLPMO ........................................ 18
Fig. S9. Spectrofluorimetric titration of apo-miniLPMO with $CuSO_4$ ........................................ 19
Fig. S10. Spectrophotometric competitive titration of $Cu^{+}$-miniLPMO with BCA ...... 20
Fig. S11. UV/Vis and CW-EPR spectra of $Cu^{2+}$-miniLPMO at different pHs ........................................ 21
Fig. S12. Vis spectra of $Cu^{2+}$-miniLPMO, at neutral, alkaline and back to neutral pH. 22
Fig. S13. Q-band EPR spectra of $Cu^{2+}$-miniLPMO at pH 4.5 and pH 11 ........................................ 23
Fig. S14. Q-band ENDOR spectra of $Cu^{2+}$-miniLPMO at pH 4.5 ........................................ 24
Fig. S15. Q-band ENDOR spectra of $Cu^{2+}$-miniLPMO at pH 11 ........................................ 25
Fig. S16. X-band HYSCORE spectrum of $Cu^{2+}$-miniLPMO at pH 4.5 ........................................ 26
Fig. S17. UV-Vis spectra of $Cu^{2+}$-miniLPMO upon treatment with $H_2O_2$ ........................................ 27
Fig. S18. Progress curve of PNPG oxidation using $Cu^{2+}$-miniLPMO or $CuSO_4$ ........................................ 28

**Tables S1 to S2**

Table S1. Comparison of the number of residues within 8 Å of the metal atom in different 4-helix bundle topologies and in representative LPMOs ........................................ 29
Table S2. $^{14}N$ spin Hamiltonian parameters of the pH dependent species employed in the simulation of the ENDOR and HYSCORE spectra ........................................ 30

**SI References ........................................ 31**

**Supporting Information Text**

**Design of miniLPMO.** The design of miniLPMO involved the following steps, here detailed.

***- Histidine brace (HB) retrostructural analysis.*** To select the best bundle topology for HB inclusion, a retrostructural analysis of HB sites has been performed on representative LPMO crystal structures. The LPMO crystal structures were retrieved from the PDB using an advanced search with the following query:

**QUERY:** ( Experimental Method = "X-RAY DIFFRACTION" **OR** Experimental Method = "NEUTRON DIFFRACTION" ) **AND** (Structure Title **HAS EXACT PHRASE** "lytic polysaccharide monooxygenase" **OR** Additional Structure Keywords **HAS EXACT PHRASE** "lytic polysaccharide monooxygenase" **OR** Additional Structure Keywords **HAS EXACT PHRASE** "LPMO" **OR** Structure Title **HAS EXACT PHRASE** "LPMO") **AND** (Chemical Name **HAS ANY OF WORDS** "copper" **OR** Chemical Name **HAS ANY OF WORDS** "zinc")

Copper and zinc were included in the query to exclude structures with an empty metal site. The resulting entries were grouped at 100% sequence identity, and the resulting 41 representative structures were downloaded for further analysis. Neighbor residue lists were extracted by selecting atoms within 8.0 angstroms of the histidine-brace site using PyMOL[1] with the following selection expression:

```
cmd.select('neighbour_Calpha', '(byres object_id near_to 8 of metal_atom) and n. CA and e. C')
```

where neighbor_Calpha is the generated selection containing the C-alpha atoms of all residues having at least one non-hydrogen atom within the threshold, object_id is the name of the loaded PDB entry and metal_atom is the selection containing the metal in the HB site. The distribution of neighboring residues has been plotted as a histogram reporting how many times a specific number of neighbors was found within the query (Fig. S1). The most likely number of neighbors corresponds to 14, as compared to the 18 residues found in the more buried type II copper site belonging to *H. rosellus* galactose oxidase (PDB ID: 1gof).

The sequences of the representative entries were aligned using MAFFT[2,3] with the L-INS-I strategy, BLOSUM62 scoring matrix, gap opening penalty 1.5 and offset value 0.14. The 15 residue positions corresponding to the metal site neighbors in PDB ID 6rw7 were identified in the alignment and used to build a logo recapitulating residue conservation in the primary and secondary coordination spheres of the metal site across the representative LPMO structures (Fig. S2). The logo was generated using the logomaker python package.[4]

This analysis points out that a few conserved residues can be considered as crucial in supporting HB structure or catalytic activity. In particular, *(i)* Gly2 is required to support the uncommon His1 conformation, *(ii)* Tyr or Asn at position 104 are highly conserved to support substrate binding, *(iii)* Gln, Glu or Asn at position 193 are involved in a H-bond interaction

with the exogenous copper-bound water, (iv) Tyr or Phe at position 195 most probably support the highly distorted copper geometry (or loosely bind copper in the case of Tyr upon substrate binding).

- ***Histidine Brace incorporation.*** To determine the most appropriate position for the inclusion of a HB site, all backbone-dependent rotamers of the internal His in AA10 LPMO from *Teredinibacter turnerae* (PDB ID: 6rw7)[5] were superimposed to all residues whose Cα was within 10 Å of the N-terminal nitrogen atom in the structure of α2D (PDB ID: 1qp6).[6] The superpositions were realized with the pair_fit function of the PyMOL software (https://www.pymol.org/).[1]
Ile22 and Val3 were identified as the ideal positions for the internal and N-terminal His, respectively. Accordingly, the first two residues of the $\alpha_2$D sequence were removed, and the N-terminal region was reconstructed to optimize His1 position relative to the newly introduced HB site.
N-terminal region reconstruction was performed using the MASTER software (https://grigoryanlab.org/master/).[7] The N-terminal His and the residues from 4 to 8 (EELEK) in the α2D sequence were used as query structure for the search. The search was performed using the default database available from the same source as the MASTER program, composed of about 17000 non redundant single chain entries extracted from the PDB. RMSD was computed over all backbone atoms, and the cutoff value for matches was fixed at 0.5 Å.
The fragments found from the MASTER search were ranked based on their RMSD from the query structure. One of the best search results was transplanted into α2D to recreate the N-terminal helix (**Fig. S3A**) while guaranteeing optimal backbone geometry for the HB site.

- ***Loop reconstruction.*** The query structure for loop reconstruction was composed of two five-residue segments from $\alpha_2$D: residues 6-10 (LEKKF) and 22-26 (IEELH). A gap of eleven residues was left between the two segments to be filled by MASTER, reconstructing the loop region (**Fig. S3B**).
For both N-terminal and loop searches, the .seq files containing the sequences of all matches and the corresponding RMSD values from the query structure were analyzed to extract the frequency of different amino acids for each position. These statistics were used during the design step to inform the allowed residues for the loop region in all ROSETTA resfiles. MASTER search matches were also used to inform the identity of allowed residues for the N-terminal segment (positions 2 – 6 of the modified sequence). The resulting modified version of $\alpha_2$D, named α2D-C4, was used as the starting structure in the Rosetta design protocol.
- ***Fixed backbone design and molecular dynamics.*** ROSETTA software (rosettacommons.org, version 3.12) was used to perform a design protocol under the Rosetta Scripts environment.[8–11] REF2015 was used as the scoring function.[12]

MD simulations (**Fig. S4**) on the lowest energy structure among all variants generated were performed with the NAMD[13,14] engine using the CHARMM36 force field.[15,16] The input files for all simulations were created using CHARMM-GUI.[17] The structures were solvated in an octahedral water box with a 10 Å edge distance. The solvent was explicitly modeled, and water molecules were described by TIP3P parametrization. Chloride or potassium atoms were placed with Monte Carlo method in order to ensure electroneutrality. Long-range electrostatic interactions were calculated using the Particle Mesh Ewald (PME) method with 1 Å grid spacing. The system was first minimized with 10000 conjugate gradient steps. Subsequently, the temperature was linearly raised from 0 to 320 K during 5 ns. The system was then equilibrated for 5 ns, and production was conducted for 10 ns in short simulations or 80 ns in extended simulations. Constant temperature was maintained through Langevin dynamics with damping coefficient 1.0 $ps^{-1}$. Constant pressure was imposed through Nose-Hoover Langevin piston with 50 fs piston period and 25 fs oscillation decay time. Integration step was set to 2 fs and all hydrogen bond lengths were fixed with the SHAKE method. VMD (Visual Molecular Dynamics)[18] was used to perform data analysis and elaboration.

**Peptide Synthesis and purification.** miniLPMO monomeric sequence was synthesized by automatic solid-phase synthesis (ABI 433A peptide synthesizer, Applied Biosystem, Foster City, CA, USA) on a 0.1 mmol scale. An H-PAL ChemMatrix resin with a substitution level of 0.2 mmol/g was used as the solid support for synthesis. Standard Fmoc synthetic protocols were used for deprotection, activation, coupling and capping cycles. After synthesis completion, the resin was washed four times with DCM, NMP, isopropanol and methanol, and finally dried.

Cleavage of the peptide from the resin with concomitant sidechain deprotection was achieved using a mixture of 95% (v/v) trifluoroacetic acid (TFA), 2.5% (v/v) water and 2.5% (v/v) triisopropylsilane (TIS), providing the peptide with amidated C-terminal. The reaction was carried out for one hour at 0° C and one hour at room temperature, under magnetic stirring. The resin was then filtered under vacuum and washed with neat TFA. The solution was concentrated to a small volume and the crude peptide was precipitated by adding an excess of cold diethyl ether. After centrifugation, the supernatant was removed, and the precipitate was washed twice with fresh diethyl ether. The peptide was dried to remove diethyl ether, re-dissolved in water and lyophilized. The yield of the crude peptide was 88% based on the resin substitution level.

The crude peptide was analyzed by analytical RP-HPLC, performed with a Shimadzu LC-10ADvp equipped with a SPDM10Avp diode-array detector. Peptide identity was assessed by high resolution mass spectrometry analysis, using a Shimadzu LCMS-IT-TOF system with ESI interface.

Purifications were accomplished by preparative RP-HPLC using a Shimadzu LC-8A connected to an SPD-20A Shimadzu UV-Vis spectrophotometric detector. Peak identity was

verified by LC-MS. The fractions containing the pure product were collected, concentrated and lyophilized.

All analyses were performed with a Vydac C18 column (2.1 mm x 100 mm; 5 μm), eluted with an $H_2O$ 0.1% v/v trifluoroacetic acid, TFA, (eluent A) and $CH_3CN$ 0.1% v/v TFA (eluent B) linear gradient, from 10 to 95% (solvent B), over 24 minutes, at 0.2 ml $min^{-1}$ flow rate. Purifications were performed using a Vydac C18 colums (22 mm x 250 cm; 10 μm) at a flow rate of 23 $mL \cdot min^{-1}$. The same solvent gradient of analytical HPLC was used, appropriately scaled up. Analytical RP-HPLC chromatogram and ESI-MS spectrum of pure miniLPMO are shown in **Fig. S5**.

**UV-Vis spectroscopy**. UV–Vis absorption spectra were recorded with a Cary Varian 60 spectrophotometer, equipped with a thermostatic cell holder (Varian, Palo Alto, CA, USA), using a quartz cuvette with a 1 cm path length. Wavelength scans were performed at 25°C from 200 to 800 nm, with a 600 nm $min^{-1}$ scan speed. Stock solutions of miniLPMO were freshly prepared by dissolving the lyophilized peptide in MilliQ water. The concentration of miniLPMO in the stock solution was determined by measuring the absorbance at 280 nm, using $\varepsilon_{280nm}$ = 5500 $M^{-1}$ $cm^{-1}$ (monomer).[19] Concentration of miniLPMO is referred to the monomeric form throughout the text, unless otherwise stated. Stock solutions of $CuSO_4$ were prepared at 10 mM concentration starting from the solid $CuSO_4 \cdot 5H_2O$. Copper concentration in the stock solutions was determined by the BCA (bicinchoninic acid) assay.[20] The latter was performed by diluting the stock solution to a final concentration of ~ 0.1 mM, then adding 5 molar equivalents of ascorbic acid and 10 molar equivalents of BCA. The solution was left to incubate for 30 min. The concentration of the $[Cu^{+}(BCA)_2]^{3-}$ complex, which is equal to the initial $Cu^{2+}$ concentration, was measured by reading the absorbance at 562 nm and using $\varepsilon_{562nm}$ = 7700 $M^{-1}$ $cm^{-1}$.[20]

In pH dependent studies, different samples of $Cu^{2+}$-miniLPMO (100 μM) in a mixed buffer solution (MES, HEPES, CHES, 15 mM each) were prepared. The complex was prepared by adding an equimolar amount of $CuSO_4$ with respect to monomer into the buffer solution containing the apo-protein. Samples from pH 2 to 11 (in 0.5 pH unit increments) were prepared by adding NaOH and $H_2SO_4$ solutions and using a pH meter to measure the final pH. These solutions were lyophilized and subsequently redissolved with MilliQ water to a final concentration of 200 μM. The reversible formation of the pH-dependent species was followed by Visible spectroscopy (**Fig. S12**), by preparing a 150 μM miniLPMO solution at pH 6.8 in the mixed buffer (see above), in the presence of an equimolar amount of $CuSO_4$. The pH of this solution was then raised at 10.8 (by NaOH addition) and finally restored at 7.3 (by $H_2SO_4$ addition).

**CD spectroscopy.** CD measurements were performed using a J-1500 spectropolarimeter equipped with a thermostatic cell holder (JASCO, Easton, MD, USA). Spectra were collected at 25°C, from 260 to 190 nm at 0.5 nm intervals with a 20 nm $min^{-1}$ scan speed, at 1 nm

bandwidth and 4 s response. Cells of 1, 0.5, and 0.2 mm pathlength were used depending on the peptide concentration. All solutions were prepared in mixture of MES, HEPES, and CHES buffer adjusted at different pH values. Samples at 100 μM miniLPMO at pH 4.5, 7.0 and 11 were prepared in the MES-HEPES-CHES mixture at 5 mM concentration of each buffer, in the absence or in the presence of 100 μM $CuSO_4$. Samples of miniLPMO at different concentrations were prepared in the MES-HEPES-CHES mixture at pH 7.4, using varying buffer concentrations (from 1.25 to 5 mM of each buffer), depending on sample concentration. The concentration of buffers was kept as low as possible to ensure the desired pH while avoiding strong absorption of the buffers in the far-UV region. CD spectra of apo-miniLPMO at different (25-100 μM) concentrations and at different pH values (4.5, 7, 11) are shown in **Fig. S6**. Superimposition of CD spectra of apo- and holo-miniLPMO at different pH values are shown in **Fig. S7**.

**Analytical Ultracentrifugation (AUC).** Sedimentation analysis was performed in the analytical ultracentrifuge ProteomeLab XL-I (Beckman Coulter, Brea, CA, USA) using an An50-Ti rotor and double-sector cells equipped with 1.5, 3, or 12 mm titanium centerpieces (Nanolytics, Potsdam, Germany), depending on the sample absorbance. Samples at 80 μM and 300 μM concentration were analyzed in the mixture of 10 mM MES, 10 mM HEPES, and 10 mM CHES at pH 4.5, 6.5, or 9.5 (**Fig. S8 A-B**). Later, samples at various concentrations were analyzed in 30 mM HEPES pH 7.5 or 30 mM HEPES with 100 mM $Na_2SO_4$ (**Fig. S8 C-E**). In the latter case, $Na_2SO_4$ was added to improve the solubility of the holo-miniLPMO species at pH 7.5. Into holo-miniLPMO samples, the appropriate volume of 10 mM $CuSO_4$ was added to match the peptide concentration in the given sample. Sedimentation velocity experiments were recorded at 20°C and 50,000 rpm as absorbance at 280 nm with 300 scans in 7-minute steps. Buffer density, viscosity, and miniLPMO partial specific volume were estimated in Sednterp.[21] Data were analyzed with Sedfit[22] using the c(s) continuous sedimentation coefficient distribution model. Figures were prepared in GUSSI.[23]

**$Cu^{2+}$ and $Cu^{+}$ binding affinities.** The binding affinity of miniLPMO towards $Cu^{2+}$ was investigated by spectrofluorimetric titrations using a FluoroMax4 (Horiba Scientific) equipped with thermostatic cell holder and magnetic stirrer. The quenching of the Trp fluorescence upon binding of $Cu^{2+}$ was followed, as previously reported in the literature.[24]

Titrations were carried out at 25°C, by adding small aliquots of freshly prepared aqueous stock solutions of $CuSO_4$ to a solution of apo-miniLPMO in 50 mM HEPES buffer at pH 7.0. Upon each addition of $CuSO_4$, the solution was stirred for 5 min to reach equilibrium, and then the fluorescence spectra were recorded.

Experiments were performed at four different peptide concentrations, ranging between 3 and 7 μM.

The fluorescence intensity at 350 nm was normalized with respect to the initial value and plotted against the total $Cu^{2+}$ concentration (**Fig. S9**). The experimental data points were fitted to a 1:1 binding isotherm arranged in the form of Eq. 1.

$$\boldsymbol{I_F = I_0 - \left(\frac{(I_0 - I_z)}{2[P]}\right)\left(([Cu^{2+}] + [P] + K_d) - \sqrt{([Cu^{2+}] + [P] + K_d)^2 - (4[P][Cu^{2+}])}\right)} \quad \text{(Eq.1)}$$

Where:

$I_F$ is the fluorescence intensity at each point during the titration;

$I_0$ is the initial fluorescence intensity (before copper addition);

$I_z$ is the fluorescence intensity value at the end of the titration;

$[Cu^{2+}]$ is the total concentration of copper in solution, varied during the titration;

[P] is the total peptide concentration, considered as monomer;

$K_d$ is the apparent dissociation constant for the $Cu^{2+}$-miniLPMO complex

$K_d$ for the $Cu^{2+}$-miniLPMO complex was derived taking into account the formation of the $Cu^{2+}$/HEPES complex (log β = 3.22).[25]

The $Cu^+$ binding affinity of miniLPMO was determined by competitive titrations, using BCA as a competitive ligand for $Cu^+$. Stock solutions of $Cu^+$ were prepared by dissolving copper(I) tetrakis(acetonitrile) hexafluorophosphate in acetonitrile, and their concentrations were determined using BCA. Titrations were performed under argon atmosphere, and all solutions were degassed by argon flushing before the experiments. Solutions of 40 μM miniLPMO and 35 μM $Cu^+$ were prepared in 50 mM HEPES buffer at pH 7.0 and treated with increasing amounts of BCA. The formation of the $[Cu^+(BCA)_2]^{3-}$ complex was followed by monitoringthe absorbance at 562 nm ($\varepsilon_{562nm}$ = 7700 $M^{-1}$ $cm^{-1}$). The concentration of the $[Cu^+(BCA)_2]^{3-}$ complex was plotted against the total BCA concentration (**Fig. S10**).

Experimental data were fitted to a ligand exchange equilibrium, arranged in the form of Eq. 2.

$$[\boldsymbol{L}]_{tot} = 2[\boldsymbol{ML_2}] + \left(\frac{([\boldsymbol{P}]_{tot}-[\boldsymbol{M}]_{tot}+[\boldsymbol{ML_2}])([\boldsymbol{ML_2}])}{\boldsymbol{K_d^P b_2}([\boldsymbol{M}]_{tot}-[\boldsymbol{ML_2}])}\right) \quad \text{(Eq.2)}$$

Where:

$[L]_{tot}$ is the total concentration of the competitive ligand (BCA), varied during the titration;

$[M]_{tot}$ is the total concentration of $Cu^+$ (fixed);

$[P]_{tot}$ is the total concentration of the protein (fixed);

$[ML_2]$ is the concentration of the $[Cu^+(BCA)_2]^{3-}$ complex, monitored during the titration;

$b_2$ is the formation constant of the $[Cu^+(BCA)_2]^{3-}$ complex ($1.6 \cdot 10^{17}$ $M^{-2}$);[26]

$K_d$ is the dissociation constant of the $Cu^+$-miniLPMO complex.

The redox potential for the $Cu^{2+}/Cu^+$-miniLPMO couple ($E^0_{miniLPMO}$) was calculated through Eq. 3.

$$\boldsymbol{E^0_{miniLPMO}} = \boldsymbol{E^0_{\frac{Cu^{2+}}{Cu^+}}} + \mathbf{0.05916 \cdot \log}\left(\frac{K_d^{Cu^{2+}}}{K_d^{Cu^+}}\right) \quad \text{(Eq. 3)}$$

Where:

$\boldsymbol{E^0_{\frac{Cu^{2+}}{Cu^+}}}$ is the standard reduction potential for the $Cu^{2+}/Cu^+$ couple (0.153 V).

$\boldsymbol{K_d^{Cu^{2+}}}$ and $\boldsymbol{K_d^{Cu^+}}$ are the dissociation constants of the $Cu^{2+}$-miniLPMO and $Cu^+$-miniLPMO complexes, respectively

**EPR spectroscopy.** X band (~ 9.44 GHz) CW-EPR experiments were performed on a Bruker EMX spectrometer equipped with a cylindrical cavity. All spectra (**Fig. S11B**) were recorded at 77 K and a microwave power of 0.683 mW, a modulation amplitude of 0.5 mT and a modulation frequency of 100 kHz. Different samples of $Cu^{2+}$-miniLPMO (100 mM) in a mixed buffer solution containing 15 mM MES, 15 mM HEPES, and 15 mM CHES were prepared. The same preparation was employed as performed with UV-Vis absorption spectroscopy (**Fig. S11A**). These solutions were lyophilized and subsequently redissolved with 30% of glycerol, used as a glassing agent, to a final concentration of 300 μM. X- and Q-band Pulse EPR experiments were performed on a Bruker Elexsys E580 spectrometer equipped with a Cryogenic cryogen-free variable temperature cryostat. During the

measurements, the resonator was overcoupled to minimize ringdown following the application of the microwave pulses.

Q-band Electron Spin Echo (ESE) detected EPR (**Fig. S13**) spectra were measured at $T = 30$ K using a Hahn echo sequence ($\pi/2$–τ–π–τ–echo) while sweeping the field with $\tau = 200$ ns.

Q-band Davies ENDOR measurements (**Fig. S14-S15**) were recorded at 30 K and carried out using the following pulse sequence: π-T-π/2-τ-π-τ-echo with $t_\pi = 32$ ns, $t_{\pi/2} = 16$ ns, and an interpulse time τ of 400 ns. The *RF* pulse length was set to 14 µs and a resolution of 440 points was adopted.

X-band six-pulse Hyperfine Sublevel Correlation experiments[27,28] (**Fig. 5 and S16**) were carried out with the extended pulse sequence $(\pi/2)_x-\tau_1-(\pi)_x-\tau_1-(\pi/2)_y-t_1-(\pi)_y-t_2-(\pi/2)_y-\tau_2-(\pi)_y-\tau_2-$echo, applying a eight-step phase cycle in order to eliminate unwanted echoes. The $t_1$ and $t_2$ time intervals were incremented in steps of 16 ns, starting from 100 ns to 4900 ns. Pulse lengths $t_{\pi/2} = 16$ ns and $t_\pi = 32$ ns, and a 0.5 kHz shot repetition rate, and equal $\tau_1$ and $\tau_2$ =136 ns values were used. The time traces of the HYSCORE spectra were baseline corrected with a third-order polynomial, apodized with a Hamming window, and zero-filled. After the two-dimensional Fourier transformation, the absolute-value spectra were calculated. All of the EPR, ENDOR, and HYSCORE simulations were performed using the Easyspin software package,[29] running within the MathWorks MATLAB environment.

**Hydrogen peroxide activation.** A solution of 100 µM $Cu^{2+}$-miniLPMO in 80 mM HEPES pH 7.7 was treated with 50 molar equivalents of $H_2O_2$ (5 mM final concentration), taken from an appropriately diluted stock solution. Changes in the UV-Vis spectra were monitored over time for 60 minutes (**Fig. S17**).

***p*-nitrophenyl-β-D-glucopyranoside (PNPG) oxidation**. Reactions were performed at different pH values (4.5, 7, 8.5, 11) in a mixed buffer composed of MES, HEPES and CHES (15 mM each), in a total volume of 500 µL. The concentration of the $Cu^{2+}$-miniLPMO was fixed at 80 µM (monomer). Given that miniLPMO forms a dimeric complex with $Cu^{2+}$ with a 2:1 copper:dimer ratio, the concentration of monomeric peptide corresponds to that of copper sites. PNPG stock solutions were freshly prepared by dissolving the neat product in MilliQ water. $H_2O_2$ stock solutions were prepared by diluting concentrated $H_2O_2$ (30% w/w) in MilliQ water, whose concentration was determined by measuring the absorbance at 240 nm ($\varepsilon_{240nm} = 43.6$ $M^{-1}cm^{-1}$).[30] The final concentration of PNPG in the reaction solution was 8 mM, and final concentration of $H_2O_2$ was 80 mM.

The reaction was started by adding of $H_2O_2$ to a solution of $Cu^{2+}$-miniLPMO and PNPG under magnetic stirring. The amount of PNP produced was determined at different times by taking an aliquot of the reaction solution and diluting it in 100 mM carbonate buffer pH 10.5.

The product was quantified by measuring the absorbance at 400 nm ($\varepsilon_{400nm}$= 18500 $M^{-1}cm^{-1}$).[31]

The reactivity of free $Cu^{2+}$ at pH 7.0 was investigated by performing the same experiment using 80 µM $CuSO_4$ in the absence of miniLPMO (**Fig. S18**).

Blank experiments were performed in the same reaction conditions, but without $Cu^{2+}$-miniLPMO. The contribution of the blank was subtracted from all the progress curves.

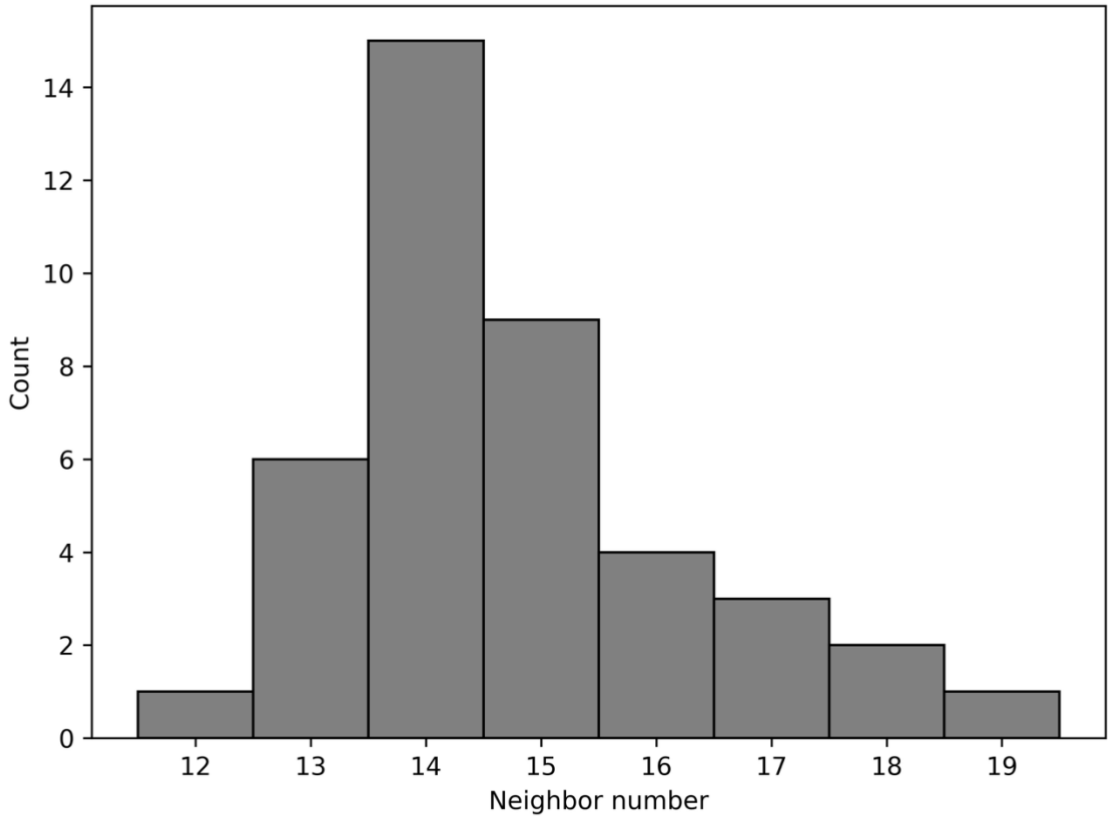


**Fig. S1**. Distribution of neighboring residue number among the 41 representative structures of LPMOs.

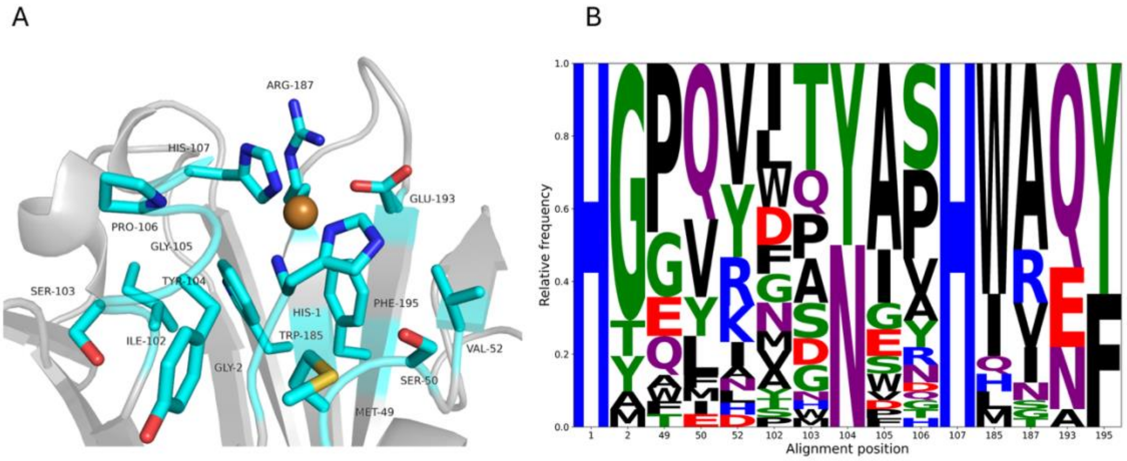


**Fig. S2**. (A) Histidine brace metal site as found in the *Tt*AA10 crystal structure (PDBID:6rw7). The 15 neighboring residues identified in 6rw7 are represented as cyan sticks. (B) Logo representing amino acid conservation of the 15 neighboring residues identified in 6rw7 extracted from a MSA containing the 41 LPMOs representative sequences extracted from the PDB. The alignment was generated using Mafft.[2]

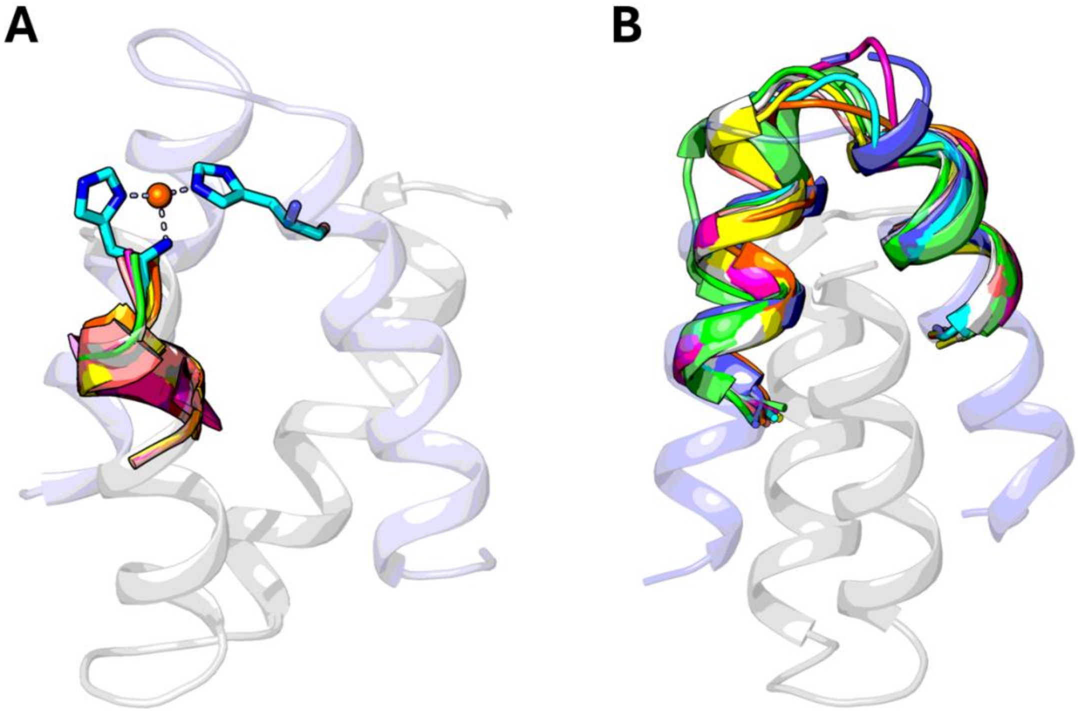


**Fig. S3**. (A) $\alpha_2$D N-terminal segment reconstruction by structural search. Only the five highest ranked matches are shown as cartoons. For simplicity, only one HB per homodimer is shown. (B) Loop redesign in $\alpha_2$D by structural search. Only the nine highest ranking matches are shown as cartoons.

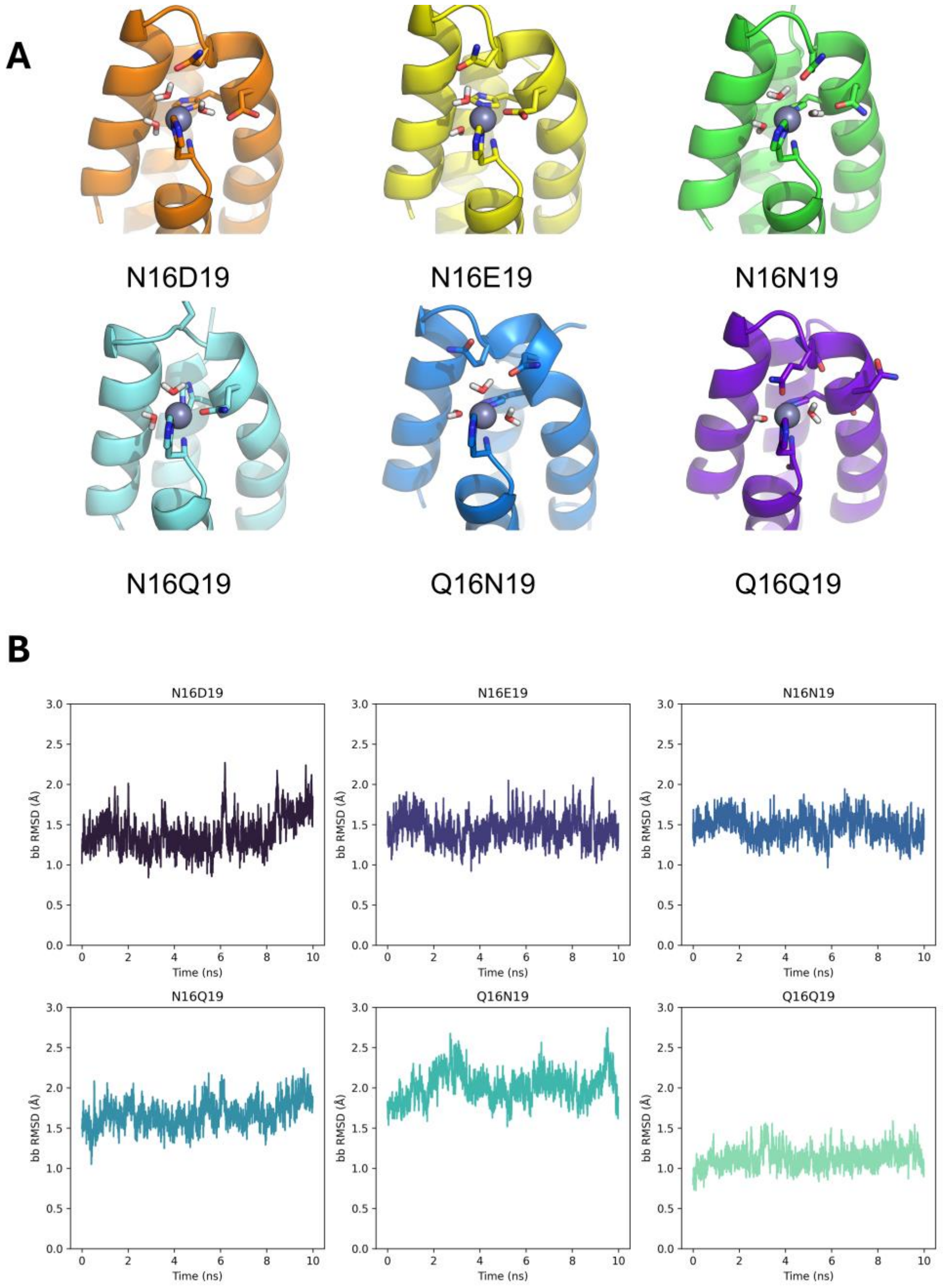


**Fig. S4.** (A) miniLPMO variants with different mutations of the secondary coordination sphere (residues 16 and 19) showing the resulting local environment and metal atom substitution. The structures shown were generated by solvating Rosetta models in an octahedral water box and performing 10000 steps of minimization on the whole system without explicitly imposing any structural restraints. (B) Backbone RMSD profiles for short MD simulations performed on the mutants shown in A, computed using the minimized ROSETTA models as reference.

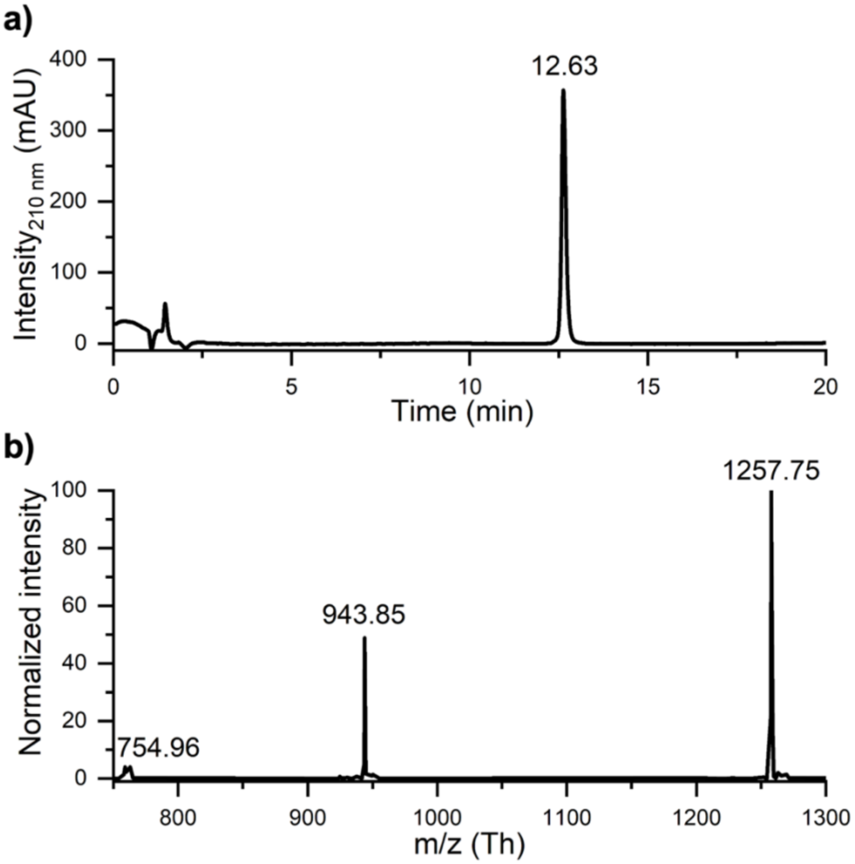


**Fig. S5.** (A) Analytical RP-HPLC chromatogram (210 nm trace) and (B) ESI-MS spectrum of pure miniLPMO. The signal at m/z = 1257.75 Th corresponds to the $[M+3H^{+}]^{3+}$ ion (theoretical average m/z: 1257.96 Th); the signal at m/z = 943.85 Th corresponds to the $[M+4H^{+}]^{4+}$ ion (theoretical average m/z: 943.72 Th); the signal at m/z = 754.96 Th corresponds to the $[M+5H^{+}]^{5+}$ ion (theoretical average m/z: 755.18 Th).

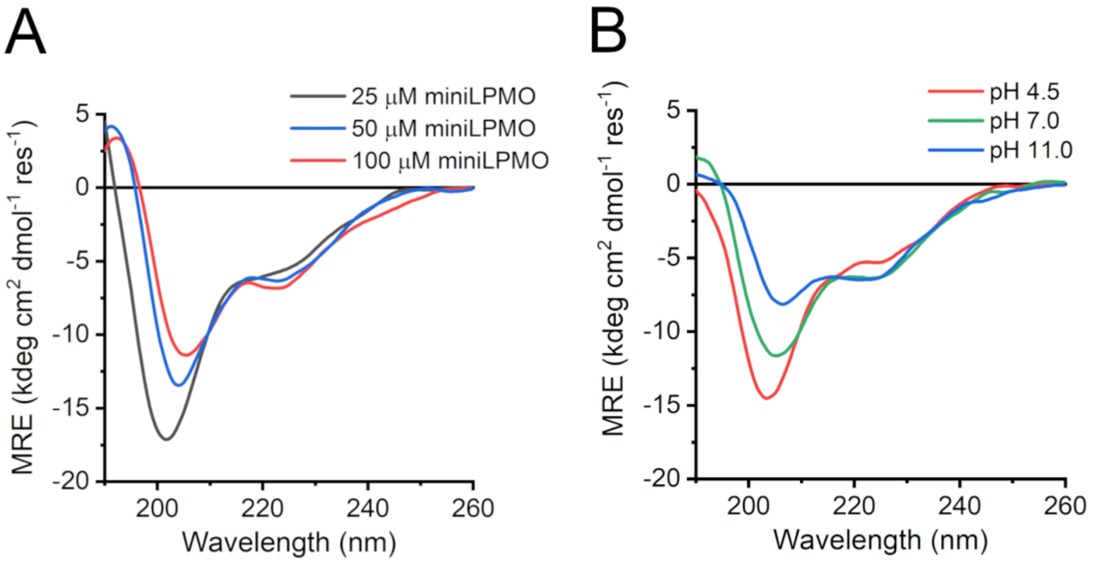


**Fig. S6.** Comparison of far UV CD spectra of apo-miniLPMO at (A) different concentrations at pH 7.5 and (B) at 100 **μ**M at different pH values.

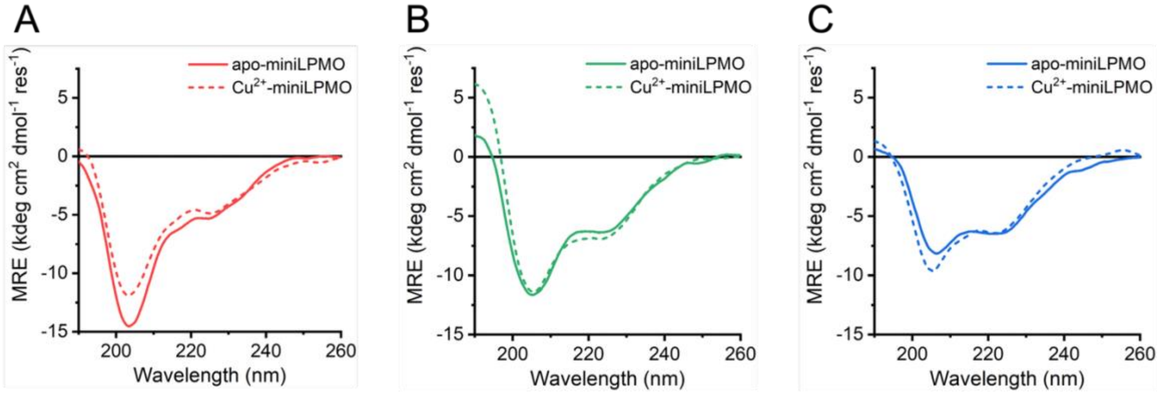


**Fig. S7.** Comparison of Far-UV CD spectra of 100 μM miniLPMO in the absence (solid lines) and in the presence (dashed lines) of 1 eq. $CuSO_4$ at (A) pH 4.5, (B) pH 7.0 and (C) pH 11.

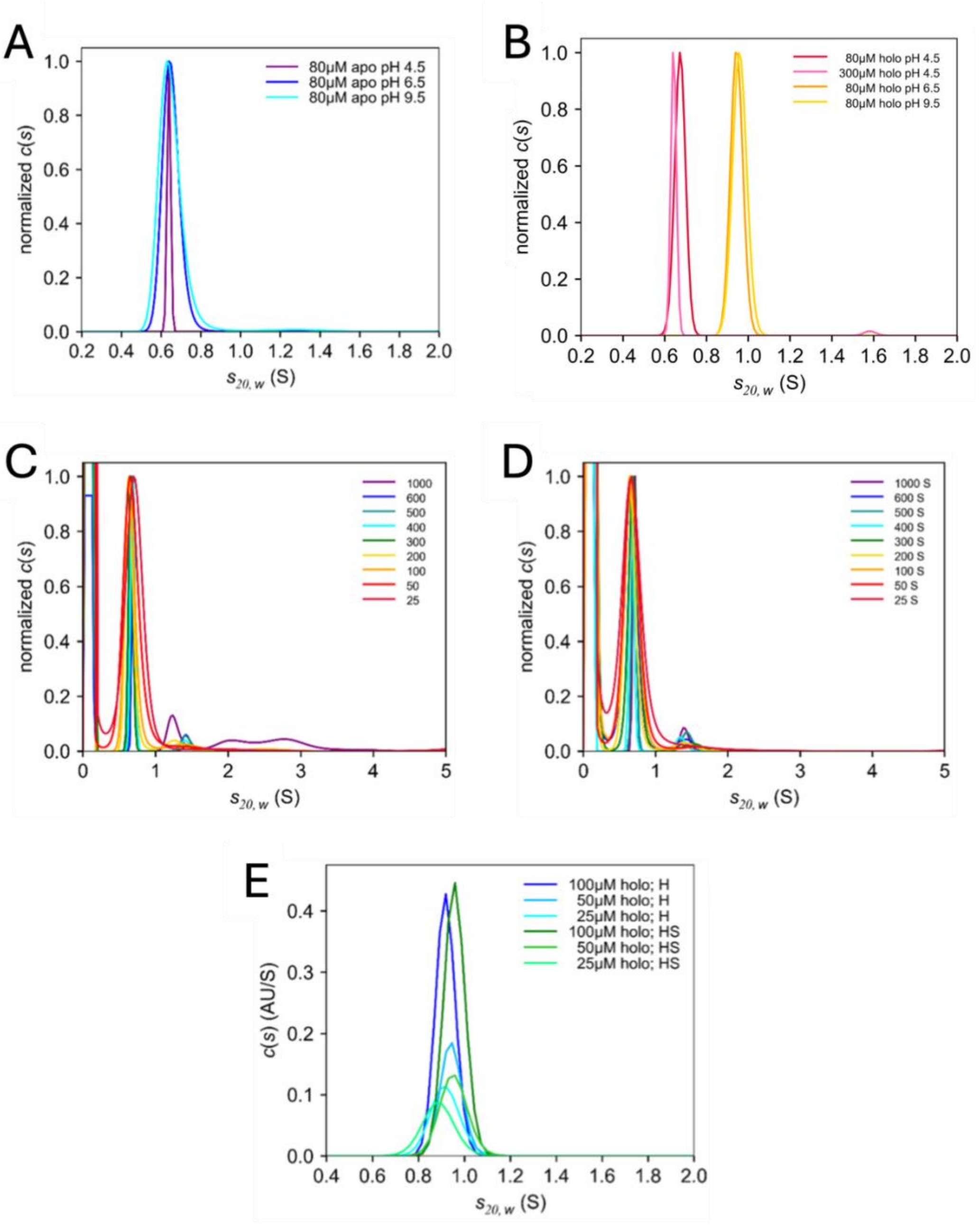


**Fig. S8.** Sedimentation velocity analysis of (A) apo- and (B) holo- miniLPMO at pH 4.5, 6.5, and 9.5 in MES-HEPES-CHES (10 mM each) buffer. Self-association of apo-miniLPMO as a function of its concentration ranging from 25 to 1000 µM analyzed at pH 7.5 in (C) 30 mM HEPES or (D) 30 mM HEPES with 100 mM $Na_2SO_4$ (S) buffer. (E) Sedimentation analysis holo-miniLPMO at concentrations from 25 to 100 µM in 30 mM HEPES (H) or 30 mM HEPES with 100 mM $Na_2SO_4$ (HS) buffer pH 7.5.

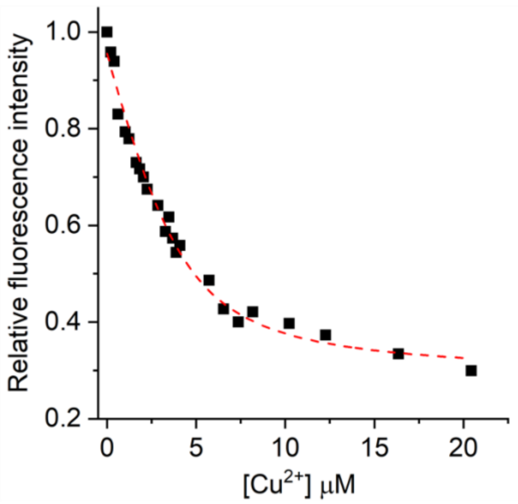


**Fig. S9.** Spectrofluorimetric titration of apo-miniLPMO (4 μM monomer) with $CuSO_4$. Experimental data points (black squares) represent relative fluorescence intensity at 350 nm. The red dashed line represents the best fitting to Eq. 1.

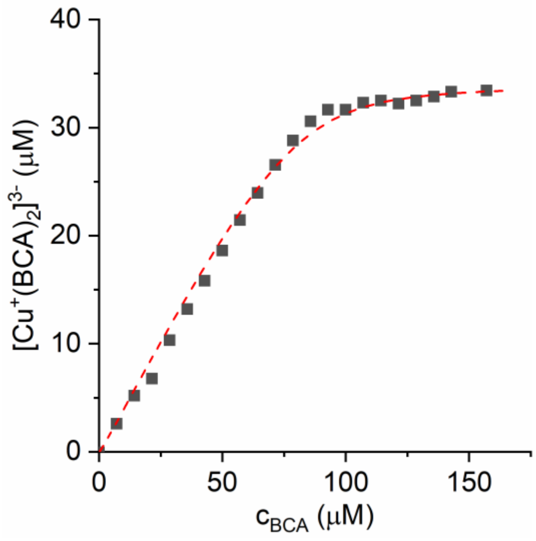


**Fig. S10.** Spectrophotometric competitive titration of $Cu^+$-miniLPMO with BCA. The red dashed line represents the best fitting to Eq. 2.

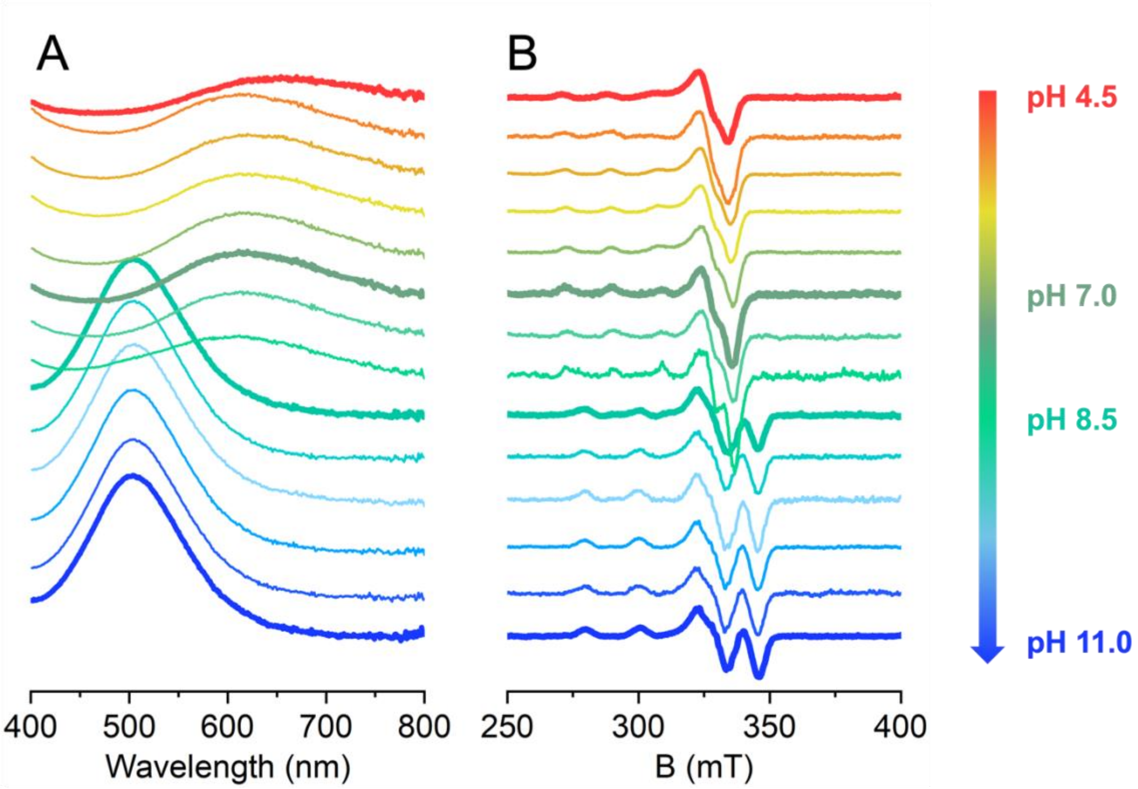


**Fig. S11.** (A) Visible absorption spectra of $Cu^{2+}$-miniLPMO (200 μM) recorded at room temperature, using a quartz cuvette with 1 cm path length and a 600 nm $min^{-1}$ scan speed. (B) X-band (9.44 GHz) CW-EPR spectra of $Cu^{2+}$-miniLPMO (300 μM) recorded at 77 K, at 25 dB attenuation (0.683 mW) 5 G modulation amplitude and 100 kHz modulation frequency. All spectra were acquired at different pH values from pH 4.5 (top) to pH 11 (bottom), with a 0.5 pH increase.

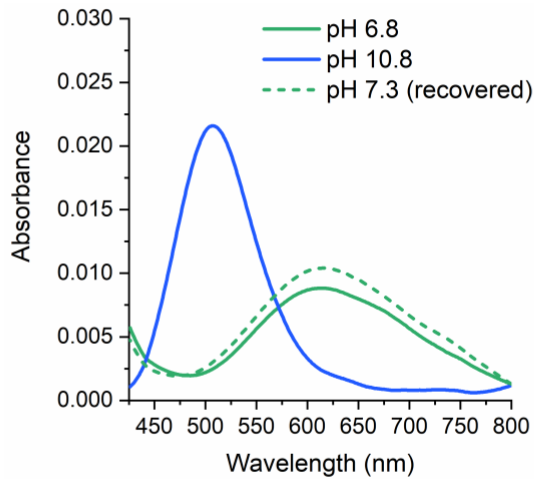


**Fig. S12**. Visible absorption spectra of $Cu^{2+}$-miniLPMO (150 μM). The solution was prepared at pH 6.8 (solid green line), then the pH was raised at 10.8 (solid blue line) and finally restored at 7.3 (dashed green line).

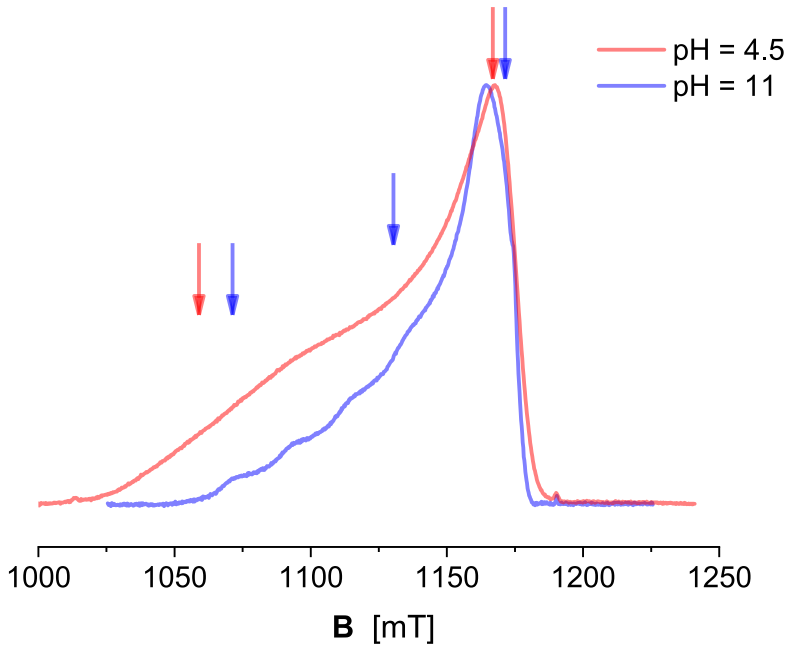


**Fig. S13.** Q-band ESE detected EPR spectra of $Cu^{2+}$-miniLPMO recorded at 20 K at pH=4.5 (red) and pH= 11 (blue). The arrows indicate the magnetic field positions at which the ENDOR spectra were recorded (Fig. S14 and Fig. S15).

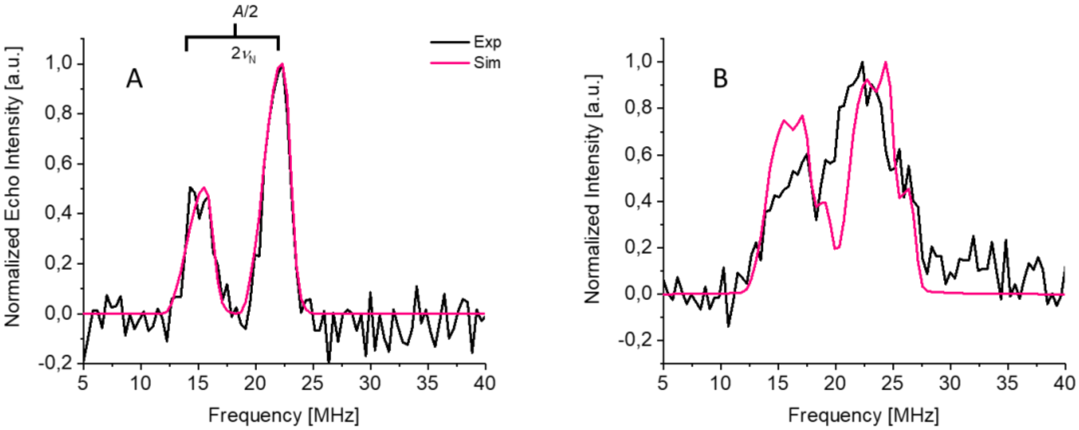


**Fig. S14.** Experimental (black) and simulated (pink) Q-band (33.77 GHz) Davies ENDOR spectra of $Cu^{2+}$-miniLPMO recorded at 20 K at pH 4.5. (A) Spectrum recorded at $B$ = 1086.1 mT (single crystal-like position), (B) spectrum recorded at $B$ = 1167.9 mT. Simulation parameters are listed in Table S1.

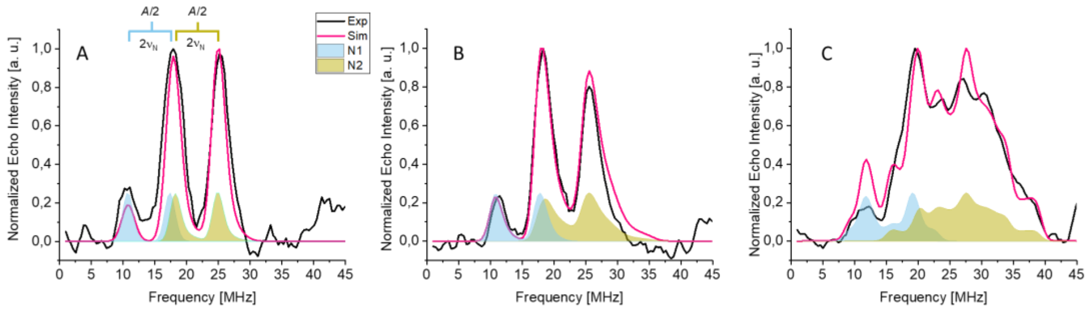


**Fig. S15.** Experimental (black) and simulated (pink) Q-band (33.77 GHz) Davies ENDOR spectra of $Cu^{2+}$-miniLPMO recorded at 20 K at pH 11 (A) spectrum recorded at $\boldsymbol{B}$ = 1087.0 mT (single crystal-like position), (B) spectrum recorded at $\boldsymbol{B}$ = 1130 mT; (C) spectrum recorded at $\boldsymbol{B}$ = 1178.0 mT. The individual contributions of the two nitrogen species are shown as shaded areas. Simulation parameters are listed in Table S1.

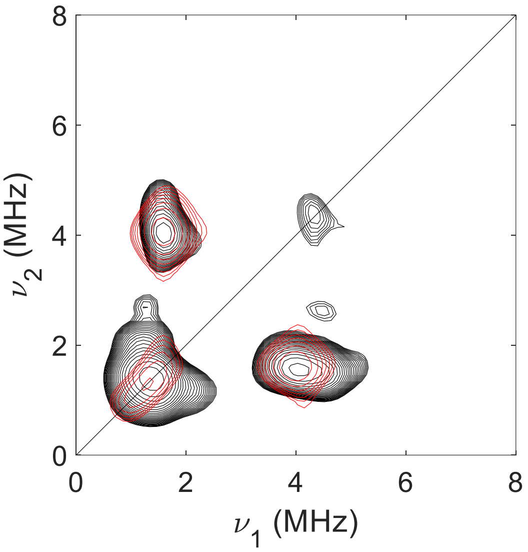


**Fig. S16.** X-band 6-pulse HYSCORE spectrum (black: experiment, red: simulation) of $Cu^{2+}$-miniLPMO at pH 4.5.

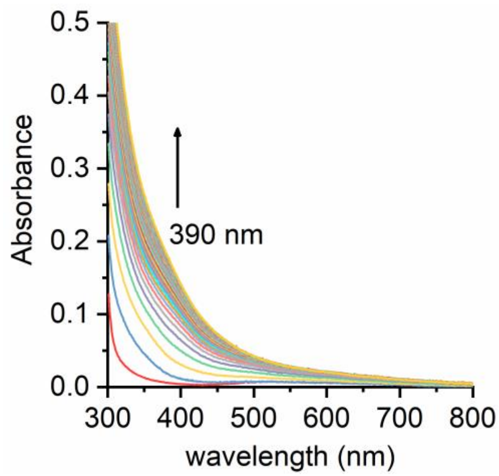


**Fig. S17.** Time dependent UV-Vis spectra acquired after addition of 5 mM $H_2O_2$ to 100 μM $Cu^{2+}$-miniLPMO at pH 7.8. Spectra were collected every minute for 60 min. The arrow indicates the increase of absorbance at 390 nm over time.

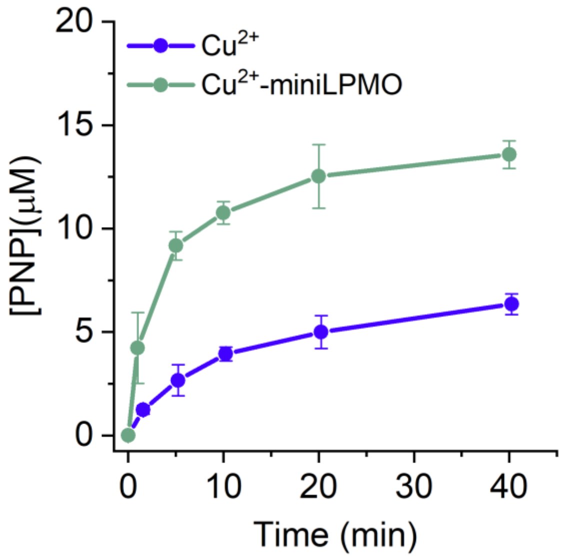


**Fig. S18.** Progress curve of PNP formation using either 80 μM $Cu^{2+}$-miniLPMO (green line) or 80 μM $CuSO_4$ (blue line) at pH 7.0. PNPG concentration was fixed at 8 mM, in 15 mM MES, 15 mM HEPES, and 15 mM CHES as a buffer. The reaction was initiated by adding $H_2O_2$ to a final concentration of 80 mM. At fixed time intervals (circles), an aliquot of the solution was taken, diluted 5 times (for $Cu^{2+}$-miniLPMO) or 2.5 times (for free $Cu^{2+}$) in 100 mM carbonate buffer pH 10.5, and PNP concentration was monitored at 400 nm.

**Table S1.** Comparison of the number of neighboring residues in different 4-helix bundle topologies and in representative crystal structures of LPMOs deposited in the PDB. The number of neighbors is defined as the total number of residues having at least one atom within 8 Å of the Cu atom. For helical bundles the metal position is obtained by superimposing the H1 of the histidine-brace site taken from AA10 (PDB ID: 6RW7) to the first residue pointing towards the interior of the bundle belonging to the N-terminal helix.

| PDB ID | Organism | Class | N° of neighbours |
|---|---|---|---|
| **Syn** | *Synthetic construct* | | 18 |
| **Anti** | *Synthetic construct* | | 16 |
| **Bisecting** | *Synthetic construct* | | 16 |
| **4B5Q** | *Phanerochaete chrysosporium* | AA9 | 13 |
| **4MAH** | *Aspergillus oryzae* | AA11 | 18 |
| **4OPB** | *Aspergillus oryzae* | AA13 | 19 |
| **4OY6** | *Streptomyces coelicolor* | AA10 | 15 |
| **4OY7** | *Streptomyces coelicolor* | AA10 | 14 |
| **4QI8** | *Neurospora crassa* | AA9 | 12 |
| **5FJQ** | *Cellvibrio japonicus* | AA10 | 16 |
| **5FOH** | *Neurospora crassa* | AA9 | 14 |
| **5FTZ** | *Streptomyces lividans* | AA10 | 14 |
| **5IJU** | *Bacillus amyloliquefaciens* | AA10 | 15 |
| **5L2V** | *Listeria monocytogenes* | AA10 | 14 |
| **5MSZ** | *Thermobia domestica* | AA15 | 15 |
| **5NNS** | *Heterobasidion irregulare* | AA9 | 14 |
| **5O2X** | *Trichoderma reesei* | AA9 | 13 |
| **5OPF** | *Micromonospora aurantiaca* | AA10 | 17 |
| **5UIZ** | *Thermobifida fusca* | AA10 | 15 |
| **5VG1** | *Jonesia denitrificans* | AA10 | 13 |
| **5WSZ** | *Bacillus thuringiensis* | AA10 | 14 |
| **6H1Z** | *Neosartorya fumigata* | AA9 | 14 |
| **6IF7**[b] | *Tectaria macrodonta* | AA10 | 14 |
| **6RW7** | *Teredinibacter turnerae* | AA10 | 15 |
| **6T5Z** | *Photorhabdus laumondii* | AA10 | 14 |
| **6YDF** | *Collariella virescens* | AA9 | 14 |
| **6YDG** | *Lentinus similis* | AA9 | 14 |
| **6Z5Y** | *Phytophthora infestans* | AA17 | 16 |
| **7EXK** | *Ceriporiopsis subvermispora* | AA9 | 13 |
| **7NTL** | *Malbranchea cinnamomea* | AA9 | 14 |
| **7OKR** | *Aliivibrio salmonicida* | AA10 | 14 |
| **7OVA** | *Neosartorya fischeri* | AA10 | 14 |
| **7PZ3** | *Thermoascus aurantiacus* | AA9 | 14 |
| **7T5D** | *Neurospora crassa* | AA10 | 15 |
| **7ZE9** | *Thermothelomyces thermophilus* | AA10 | 16 |
| **7ZJB** | *Streptomyces coelicolor* | AA10 | 15 |
| **8B7P** | *Emericella nidulans* | AA10 | 13 |
| **8CC3** | *Vibrio cholerae* | AA10 | 13 |
| **8GUL** | *Vibrio campbellii* | AA10 | 15 |
| **8RRY** | *Serratia marcescens* | AA10 | 15 |
| **8S6S**[a] | *Phytophthora sojae* | AA7 | 17 |
| **8S71**[a] | *Phytophthora sojae* | AA7 | 18 |
| **9EXJ** | *Aspergillus fumigatus* | AA10 | 17 |
| **9FDL** | *Thermothelomyces thermophilus* | AA10 | 16 |

[a] AA7 are structurally homologous to LPMOs, but they are not classified as LPMOs.

**Table S2.** $^{14}N$ spin Hamiltonian parameters of the pH dependent species employed in the simulation of the ENDOR and HYSCORE spectra reported in Figs S10, S11 and S12. Hyperfine and *e2Qq*/h values are given in units of MHz. The estimated uncertainty in the determination of the hyperfine tensor for N1-N3 is of the order of 1 MHz, while for N4 is 0.3 MHz. For the e2Qq/h an uncertainty of 0.5 MHz is estimated while for the parameter $\eta$ the uncertainty is ± 0.2.

| **Species (pH)** | **1 (pH 4.5)** | | | | | **2 (pH 11)** | | | | |
|---|---|---|---|---|---|---|---|---|---|---|
| | $\|A_1\|$ | $\|A_2\|$ | $\|A_3\|$ | $e^2Qq$/h | $h$ | $\|A_1\|$ | $\|A_2\|$ | $\|A_3\|$ | $e^2Qq$/h | $h$ |
| $N_1$ | 45 | 36 | 36 | 2.2 | 0.2 | | | | | |
| $N_2$ | | | | | | 43 | 28 | 28 | 2.2 | 0.2 |
| $N_3$ | | | | | | 67 | 43 | 43 | 2.8 | 0.5 |
| $N_4$ | 1.6 | 1.6 | 2.4 | 1.5 | 0.9 | 1.6 | 1.6 | 2.4 | 1.5 | 0.9 |

## SI References


(1) Schrödinger, LLC. The PyMOL Molecular Graphics System, Version 1.8, 2015.

(2) Katoh, K.; Rozewicki, J.; Yamada, K. D. MAFFT Online Service: Multiple Sequence Alignment, Interactive Sequence Choice and Visualization. *Brief. Bioinform.* **2019**, *20* (4), 1160–1166.

(3) Kuraku, S.; Zmasek, C. M.; Nishimura, O.; Katoh, K. aLeaves Facilitates On-Demand Exploration of Metazoan Gene Family Trees on MAFFT Sequence Alignment Server with Enhanced Interactivity. *Nucleic Acids Res.* **2013**, *41* (W1), W22–W28.

(4) Tareen, A.; Kinney, J. B. Logomaker: Beautiful Sequence Logos in Python. *Bioinformatics* **2020**, *36* (7), 2272–2274.

(5) Fowler, Claire. A.; Sabbadin, F.; Ciano, L.; Hemsworth, G. R.; Elias, L.; Bruce, N.; McQueen-Mason, S.; Davies, G. J.; Walton, P. H. Discovery, Activity and Characterisation of an AA10 Lytic Polysaccharide Oxygenase from the Shipworm Symbiont Teredinibacter Turnerae. *Biotechnol. Biofuels* **2019**, *12* (1), 232.

(6) Hill, R. B.; DeGrado, W. F. Solution Structure of α2D, a Nativelike de Novo Designed Protein. *J. Am. Chem. Soc.* **1998**, *120* (6), 1138–1145.

(7) Zhou, J.; Grigoryan, G. Rapid Search for Tertiary Fragments Reveals Protein Sequence–Structure Relationships. *Protein Sci.* **2015**, *24* (4), 508–524.

(8) Leaver-Fay, A.; Tyka, M.; Lewis, S. M.; Lange, O. F.; Thompson, J.; Jacak, R.; Kaufman, K. W.; Renfrew, P. D.; Smith, C. A.; Sheffler, W.; Davis, I. W.; Cooper, S.; Treuille, A.; Mandell, D. J.; Richter, F.; Ban, Y.-E. A.; Fleishman, S. J.; Corn, J. E.; Kim, D. E.; Lyskov, S.; Berrondo, M.; Mentzer, S.; Popović, Z.; Havranek, J. J.; Karanicolas, J.; Das, R.; Meiler, J.; Kortemme, T.; Gray, J. J.; Kuhlman, B.; Baker, D.; Bradley, P. Rosetta3. In *Methods in Enzymology*; Johnson, M. L., Brand, L., Eds.; Computer Methods, Part C; Academic Press, 2011; Vol. 487, pp 545–574.

(9) Fleishman, S. J.; Leaver-Fay, A.; Corn, J. E.; Strauch, E.-M.; Khare, S. D.; Koga, N.; Ashworth, J.; Murphy, P.; Richter, F.; Lemmon, G.; Meiler, J.; Baker, D. RosettaScripts: A Scripting Language Interface to the Rosetta Macromolecular Modeling Suite. *PLOS ONE* **2011**, *6* (6), e20161.

(10) DiMaio, F.; Leaver-Fay, A.; Bradley, P.; Baker, D.; André, I. Modeling Symmetric Macromolecular Structures in Rosetta3. *PLoS ONE* **2011**, *6* (6), e20450.

(11) Park, H.; Bradley, P.; Greisen, P. Jr.; Liu, Y.; Mulligan, V. K.; Kim, D. E.; Baker, D.; DiMaio, F. Simultaneous Optimization of Biomolecular Energy Functions on Features from Small Molecules and Macromolecules. *J. Chem. Theory Comput.* **2016**, *12* (12), 6201–6212.

(12) Alford, R. F.; Leaver-Fay, A.; Jeliazkov, J. R.; O'Meara, M. J.; DiMaio, F. P.; Park, H.; Shapovalov, M. V.; Renfrew, P. D.; Mulligan, V. K.; Kappel, K.; Labonte, J. W.; Pacella, M. S.; Bonneau, R.; Bradley, P.; Dunbrack, R. L. Jr.; Das, R.; Baker, D.; Kuhlman, B.; Kortemme, T.; Gray, J. J. The Rosetta All-Atom Energy Function for Macromolecular Modeling and Design. *J. Chem. Theory Comput.* **2017**, *13* (6), 3031–3048.

(13) Kalé, L.; Skeel, R.; Bhandarkar, M.; Brunner, R.; Gursoy, A.; Krawetz, N.; Phillips, J.; Shinozaki, A.; Varadarajan, K.; Schulten, K. NAMD2: Greater Scalability for Parallel Molecular Dynamics. *J. Comput. Phys.* **1999**, *151* (1), 283–312.

(14) Phillips, J. C.; Braun, R.; Wang, W.; Gumbart, J.; Tajkhorshid, E.; Villa, E.; Chipot, C.; Skeel, R. D.; Kalé, L.; Schulten, K. Scalable Molecular Dynamics with NAMD. *J. Comput. Chem.* **2005**, *26* (16), 1781–1802.

(15) Brooks, B. R.; Brooks, C. L.; Mackerell, A. D.; Nilsson, L.; Petrella, R. J.; Roux, B.; Won, Y.; Archontis, G.; Bartels, C.; Boresch, S.; Caflisch, A.; Caves, L.; Cui, Q.; Dinner, A. R.; Feig, M.; Fischer, S.; Gao, J.; Hodoscek, M.; Im, W.; Kuczera, K.; Lazaridis, T.; Ma, J.; Ovchinnikov, V.; Paci, E.; Pastor, R. W.; Post, C. B.; Pu, J. Z.; Schaefer, M.; Tidor, B.;

Venable, R. M.; Woodcock, H. L.; Wu, X.; Yang, W.; York, D. M.; Karplus, M. CHARMM: The Biomolecular Simulation Program. *J. Comput. Chem.* **2009**, *30* (10), 1545–1614.

(16) Lee, J.; Cheng, X.; Swails, J. M.; Yeom, M. S.; Eastman, P. K.; Lemkul, J. A.; Wei, S.; Buckner, J.; Jeong, J. C.; Qi, Y.; Jo, S.; Pande, V. S.; Case, D. A.; Brooks, C. L. I.; MacKerell, A. D. Jr.; Klauda, J. B.; Im, W. CHARMM-GUI Input Generator for NAMD, GROMACS, AMBER, OpenMM, and CHARMM/OpenMM Simulations Using the CHARMM36 Additive Force Field. *J. Chem. Theory Comput.* **2016**, *12* (1), 405–413.

(17) Jo, S.; Kim, T.; Iyer, V. G.; Im, W. CHARMM-GUI: A Web-Based Graphical User Interface for CHARMM. *J. Comput. Chem.* **2008**, *29* (11), 1859–1865.

(18) Humphrey, W.; Dalke, A.; Schulten, K. VMD: Visual Molecular Dynamics. *J. Mol. Graph.* **1996**, *14* (1), 33–38.

(19) Pace, C. N.; Vajdos, F.; Fee, L.; Grimsley, G.; Gray, T. How to Measure and Predict the Molar Absorption Coefficient of a Protein. *Protein Sci.* **1995**, *4* (11), 2411–2423.

(20) Brenner, A. J.; Harris, E. D. A Quantitative Test for Copper Using Bicinchoninic Acid. *Anal. Biochem.* **1995**, *226* (1), 80–84.

(21) Philo, J. S. SEDNTERP: A Calculation and Database Utility to Aid Interpretation of Analytical Ultracentrifugation and Light Scattering Data. *Eur. Biophys. J.* **2023**, *52* (4), 233–266.

(22) Schuck, P. Size-Distribution Analysis of Macromolecules by Sedimentation Velocity Ultracentrifugation and Lamm Equation Modeling. *Biophys. J.* **2000**, *78* (3), 1606–1619.

(23) Brautigam, C. A. Chapter Five - Calculations and Publication-Quality Illustrations for Analytical Ultracentrifugation Data. In *Methods in Enzymology*; Cole, J. L., Ed.; Analytical Ultracentrifugation; Academic Press, 2015; Vol. 562, pp 109–133.

(24) Pitts, W. C.; Deb, A.; Penner-Hahn, J. E.; Pecoraro, V. L. Revving up a Designed Copper Nitrite Reductase Using Noncoded Active Site Ligands. *ACS Catal.* **2024**, *14* (6), 4362–4368.

(25) Tegoni, M.; Yu, F.; Bersellini, M.; Penner-Hahn, J. E.; Pecoraro, V. L. Designing a Functional Type 2 Copper Center That Has Nitrite Reductase Activity within α-Helical Coiled Coils. *Proc. Natl. Acad. Sci.* **2012**, *109* (52), 21234–21239.

(26) Young, T. R.; Xiao, Z. Principles and Practice of Determining Metal–Protein Affinities. *Biochem. J.* **2021**, *478* (5), 1085–1116.

(27) Song, R.; Zhong, Y. C.; Noble, C. J.; Pilbrow, J. R.; Hutton, D. R. A New Six-Pulse Two-Dimensional Electron Spin Echo Envelope Modulation (ESEEM) Correlation Spectroscopy. *Chem. Phys. Lett.* **1995**, *237* (1), 86–90.

(28) Kasumaj, B.; Stoll, S. 5- and 6-Pulse Electron Spin Echo Envelope Modulation (ESEEM) of Multi-Nuclear Spin Systems. *J. Magn. Reson.* **2008**, *190* (2), 233–247.

(29) Stoll, S.; Schweiger, A. EasySpin, a Comprehensive Software Package for Spectral Simulation and Analysis in EPR. *J. Magn. Reson.* **2006**, *178* (1), 42–55.

(30) Noble, R. W.; Gibson, Q. H. The Reaction of Ferrous Horseradish Peroxidase with Hydrogen Peroxide. *J. Biol. Chem.* **1970**, *245* (9), 2409–2413.

(31) Selmeczi, K.; Giorgi, M.; Speier, G.; Farkas, E.; Réglier, M. Mono- versus Binuclear Copper(II) Complexes in Phosphodiester Hydrolysis. *Eur. J. Inorg. Chem.* **2006**, *2006* (5), 1022–1031.
